# Injectable and Gellable Chitosan Formulations Filled with Cellulose Nanofibers for Intervertebral Disc Tissue Engineering

**DOI:** 10.3390/polym10111202

**Published:** 2018-10-27

**Authors:** Ingo Doench, Maria E. W. Torres-Ramos, Alexandra Montembault, Paula Nunes de Oliveira, Celia Halimi, Eric Viguier, Laurent Heux, Robin Siadous, Rossana M. S. M. Thiré, Anayancy Osorio-Madrazo

**Affiliations:** 1 Institute of Microsystems Engineering IMTEK, Laboratory for Sensors, University of Freiburg, 79110 Freiburg, Germany; ingo.doench@imtek.uni-freiburg.de (I.D.); dudawtr@hotmail.com (M.E.W.T.-R.); 2 Freiburg Materials Research Center FMF, University of Freiburg, 79104 Freiburg, Germany; 3 Ingénierie des Matériaux Polymères (IMP), CNRS UMR 5223, Université Claude Bernard Lyon 1, Université de Lyon, 69622 Villeurbanne Cedex, France; alexandra.montembault@univ-lyon1.fr (A.M.); paulaoliveiraqmc@gmail.com (P.N.d.O.); celia.halimi@hotmail.fr (C.H.); 4 VetAgro Sup, Veterinary School, University of Lyon, 69280 Marcy l’Etoile, France; eric.viguier@vetagro-sup.fr; 5 Centre de Recherches sur les Macromolécules Végétales (CERMAV)-CNRS UPR 5301 Université Grenoble-Alpes, 38041 Grenoble, France; laurent.heux@cermav.cnrs.fr; 6 INSERM U1026 Bioingénierie tissulaire, Université Bordeaux, 33000 Bordeaux, France; robin.siadous@inserm.fr; 7 COPPE/Program of Metallurgical and Materials Engineering, Federal University of Rio de Janeiro, P.O. Box 68505, 21941-972 Rio de Janeiro, Brazil; rossana@metalmat.ufrj.br

**Keywords:** chitosan, cellulose nanofibers, composite hydrogel, injectable biomaterial, rheological behavior, intervertebral disc, tissue engineering

## Abstract

The development of non-cellularized injectable suspensions of viscous chitosan (CHI) solutions (1.7–3.3% (*w*/*w*)), filled with cellulose nanofibers (CNF) (0.02–0.6% (*w*/*w*)) of the type nanofibrillated cellulose, was proposed for viscosupplementation of the intervertebral disc nucleus pulposus tissue. The achievement of CNF/CHI formulations which can gel in situ at the disc injection site constitutes a minimally-invasive approach to restore damaged/degenerated discs. We studied physico-chemical aspects of the sol and gel states of the CNF/CHI formulations, including the rheological behavior in relation to injectability (sol state) and fiber mechanical reinforcement (gel state). CNF-CHI interactions could be evidenced by a double flow behavior due to the relaxation of the CHI polymer chains and those interacting with the CNFs. At high shear rates resembling the injection conditions with needles commonly used in surgical treatments, both the reference CHI viscous solutions and those filled with CNFs exhibited similar rheological behavior. The neutralization of the flowing and weakly acidic CNF/CHI suspensions yielded composite hydrogels in which the nanofibers reinforced the CHI matrix. We performed evaluations in relation to the biomedical application, such as the effect of the intradiscal injection of the CNF/CHI formulation in pig and rabbit spine models on disc biomechanics. We showed that the injectable formulations became hydrogels in situ after intradiscal gelation, due to CHI neutralization occurring in contact with the body fluids. No leakage of the injectate through the injection canal was observed and the gelled formulation restored the disc height and loss of mechanical properties, which is commonly related to disc degeneration.

## 1. Introduction

Many biological materials consist of composite hydrogels reinforced by fibers, such as the extracellular matrix (ECM) of many mammal tissues. There is increasing interest in developing bioinspired fiber-filled hydrogels for tissue engineering. In situ gelation within the body tissue is attractive, but requires an adequate design with choice of compounds able to flow, be injectable and achieve desired tissue engineered functionality. Intervertebral disc (IVD) afflictions commonly due to disc degeneration is thought to be the leading cause of low back pain (LBP) [1]. Satisfactory treatments have not been established yet [2], with the “gold standard” being the disc excision and fusion of the adjacent vertebral bodies (VBs). This may lead to further degeneration due to altered segmental motion. Strategies of disc replacement with biomaterials are available, but the surgery is invasive and the long-term beneficial effect of the prosthesis is debated [3]. The support of IVD self-regeneration constitutes a novel strategy. However, self-regeneration is complicated since the IVD is the largest avascular tissue of the human body [4,5]. The disc connects the VBs while keeping the spine movable. It is composed of three distinct tissues: the nucleus pulposus (NP), the annulus fibrosus (AF) and the vertebral endplates (VEPs). The NP is a hydrated gelatinous core enclosed by the AF [6]. Extracellular matrix (ECM)—i.e., proteoglycan (PG) hydrogel reinforced by collagen fibers—comprises 95 wt % of the IVD [6]. The water content is related to the PG content in the disc, with the highest hydration in the NP (~80–90 wt %) and a lower hydration in the AF (~65 wt %). Thus, the disc is able to withstand high compression loads and plays a key role in the spine biomechanics [7,8,9]. The NP exerts a turgor, which acts as a hydrostatic pressure to support the applied load [8,9]. The AF acts as a thick-walled pressure vessel to contain the internal pressure of the nucleus [8]. With disc degeneration, the collagen and PG composition is altered, and the water content of the nucleus falls by 10–15% [10]. When loading above 200 N is applied, for example, 20% of the disc water is driven out and the nuclear pressure falls by 36%. Roughley et al. [11] studied the suitability of chitosan/glycerophosphate/hydroxyethyl cellulose hydrogels for the encapsulation of IVD cells and accumulation of functional ECM, mimicking that of the nucleus. Nuclear supplementation has been commonly aimed against mechanical failure unless a bioactive material also could be found to promote cell growth, PG production, and disc nutrition. The treatment of the nucleus with a photocrosslinked hyaluronic acid (HA) hydrogel was proposed, which aimed to fill the NP and yield in situ a resistant hydrogel [12]. The NP augmentation by a gel-like formulation could prevent disc height loss, and biomechanical and biochemical changes associated with degeneration. Gellable materials could be injected via a minimally invasive access. The sol/gel material could integrate within the disc nucleus and act as a tissue substitute.

The aim of this work is the development and evaluation of a bioinspired injectable and gellable biomaterial, consisting of chitosan (CHI) viscous solution filled with cellulose nanofibers (CNF) for IVD repair and regeneration through the augmentation of the NP. In addition, CHI should serve as biocompatible cell growth-promoting compound, and CNFs should provide mechanical reinforcement reinstating the biomechanical properties of collagen fibrils in the disc. Chitosan structurally belongs to the glycosaminoglycan (GAG) family of polysaccharides [13]. It is the main derivative of chitin, which is mainly extracted from crustacean shells and endoskeleton of cephalopods. A large number of in vitro and in vivo studies highlight CHI biological properties, such as biodegradation [14], non-toxicity [15], cytocompatibility [16], and hemostatic activity [15]. Its exceptional biocompatibility together with its bioactivity explain the great potential of CHI for tissue engineering [14,16,17,18,19,20]. Montembault et al. [20] developed cartilage tissue enginnering CHI hydrogels whose interaction with chondrocyte cells promoted ECM production in vitro. Ladet et al. [21] prepared onion-like CHI multimembrane hydrogels, suitable for engineering of multilayer tissues, such as intervertebral disc, skin and blood vessels [19,21]. There is an increasing interest in using cellulose nanofibers (CNFs) as a renewable and environmentally-friendly reinforcement [22,23,24,25,26]. CNFs are highly available in plant biomass [26] and constitute an outstanding nanoreinforcement because of their high crystallinity and aspect ratio [22,27,28]. There are two major families of native CNFs [26,29]: the cellulose nanowhiskers (CNWs) with the highest strength and stiffness (Young’s modulus: 114–140 GPa) [30]; and the nano/microfibrillated cellulose (MFC) resulting from the peeling of the native fibers into a network of hairy fibrils [29]. Filling of CHI matrix by polysaccharide nanofibers has been mainly reported in the development of dry film or scaffold nanocomposites with enhancement of the mechanical, thermal and barrier properties [31,32,33,34,35,36,37,38]. In many of these works, instead of using unmodified cellulose fibers, the nanofibers were modified by 2,2,6,6-tetramethylpiperidine-1-oxyl radical (TEMPO) oxidation to improve the chitosan/fiber interaction [31,32]. Casting films of blends of CHI and poly(vinyl alcohol) (PVA) were also reinforced with TEMPO-CNFs (TOCNF) [31]. Some works reported the positive effect of ultrasound irradiation on the dispersion of CNFs when mixing with CHI to produce cast films [32,37]. As a nanomaterial, the question of CNF toxicity has been addressed. Low toxicity risk potential for CNFs has been reported according to ecotoxicological, cytotoxicity and pro-inflammatory response studies [39,40]. In vitro tests carried out with human and murine cells demonstrated the absence of cytotoxicity and genotoxicity for MFC suspensions [41]. Thus, CNFs have been used as biomaterial reinforcement in tissue engineering [42,43]. In hydrogel biomaterials, the use of CNFs is promising: in addition to their mechanical performance, the CNFs are biocompatible, present high water retention and could yield transparent biomaterials [42,44]. Moreover, CNFs can be oriented within hydrogels, by uniaxial stretching their composites under controlled environmental humidity. Osorio-Madrazo et al. [22] performed pioneering works where CNFs, specifically nanowhiskers, were oriented in bulk hydrogel matrices to produce reinforced anisotropic hydrogels. Thermosensitive injectable hydrogels of CHI containing TOCNF at concentrations 0.2%, 0.4%, 0.6%, and 0.8% (*w*/*v*) were designed for biomedical applications [45]. The hydrogels underwent sol-gel transition at body temperature through interactions between chitosan and β-glycerophosphate. The addition of TOCNF reduced gelation time and increased porosity. The hydrogels showed improved biocompatibility, both in vitro and in vivo, compared to CHI hydrogel. Both MC3T3-E1 pre-osteoblast cells and L929 fibroblast cells showed biocompatibility towards CHI/TOCNF 0.4% (*w*/*v*) [45]. The ability of CHI solutions to form physical hydrogels at pH approximately above 6, the outstanding mechanical performance of the CNFs, and the biological properties of both polysaccharides justify our choice for combining CNFs and CHI for the development of CNF/CHI formulations for IVD tissue engineering, as proposed in this work. These formulations should be injectable and able to gel in situ in the tissue environment without addition of any chemical crosslinker [46]. To assess the suitability of these formulations for this application, we performed investigations focused on injectability, achievement of in situ gelation within the nucleus pulposus, and viscoelastic behavior of the achieved composite hydrogels. Different parameters, such as the CHI concentration (Cp), CNF content and processing solvent conditions, were varied in the formulation design and their impact on the biomaterial properties were studied.

## 2. Materials and Method

### 2.1. Chitosan

Chitosan (Type: CHITOSAN 144, Batch No. 20120926) from squid pen chitin was supplied by Mahtani Chitosan (Veraval, Gujarat, India). The CHI degree of acetylation (DA) was 2.5%. It was determined by H^1^NMR spectroscopy following the methodology of Hirai et al. [45,46]. The measurement was performed on a Bruker ALS 300 spectrometer (Bruker GmbH, Ettlingen, Germany) (300 MHz for ^1^H) at 298 K. The number (M_n_) and weight-average molecular weight (M_w_) of CHI were 4.10 × 10^5^ g/mol (± 6.4%) and 6.11 × 10^5^ g/mol (± 9.6%), respectively, with a polydispersity index Ip = 1.49 (± 11.6%). The CHI molecular weight was determined by size exclusion chromatography (SEC) coupled to multi-angle laser light scattering (MALLS) as previously described [46].

### 2.2. Cellulose Nanofibers

Gel-like suspensions of nanofibrillated cellulose (CNF) were obtained from bleached pine sulfite dissolving pulp at the Centre Technique du Papier (CTP, Grenoble, France) by a mechano-enzymatic method adapted from Pääkkö et al. (2007) [47]. Before 1 h incubation at 50 °C with a solution of endoglucanase FiberCare R^®^ (Novozymes Biologicals, Paris, France) at pH5.0, the pulp was refined at 4.5% consistency with a 12” single disk refiner for 25 min. The digested samples were further refined to obtain a pulp suspension of SR (Schopper-Riegler) number higher than 80 and mean fiber length smaller than 300 µm. A quantity of 2% (*w*/*w*) fiber suspensions were processed with an Ariete homogenizer, involving one pass at 1000 bar followed by 3 passes at 1500 bars. The obtained CNFs displayed a surface charge density of 40–80 mmol/kg and were weakly charged with carboxylate moieties. The morphology of the cellulose nanofibers was characterized by atomic force microscopy (AFM). A droplet of a 0.001% CNF suspension was placed on a freshly cleaved mica surface. After sample drying, the observation was performed in tapping mode with an AFM (NanoScope V Controller and NanoScope 9.1 Software, Bruker Corporation, Santa Barbara, CA, USA) equipped with a tube scanner from Veeco Digital Instruments (Santa Barbara, CA, USA), using silicon tips (PPP-NCH, Nanosensors, Sindelfingen, Germany) with resonance frequency and spring constant of 360 kHz and 50 N·m^−1^, respectively. Height and phase images were analyzed with NanoScope Analysis 1.5 software.

### 2.3. Preparation of the CNF Filled-Chitosan Suspensions and Composite Hydrogels

As previously used for pure CHI solutions and hydrogels [48,49], two methods were used to prepare the CNF-filled CHI suspensions, which were subsequently used to prepare CNF/CHI composite hydrogels. In one method, the suspensions were prepared in a weakly acidic aqueous medium. In the other method, they were prepared in weakly acidic hydroalcoholic conditions. A fine powder of chitosan (CHI) was mixed at 1.7%, 2.0% or 3.3% (*w*/*w*) with CNFs in water at a given CNF content (e.g., 0.2%; 0.3%; 0.4%; 0.6% (*w*/*w*)). The dispersions were sonicated with a SONOPULS Ultrasonic homogenizer (Bandelin electronic GmbH, Berlin, Germany) for 5 min at 40% amplitude. Afterwards, acetic acid was added in stoichiometric ratio to solubilize the chitosan (DA = 2.5%) and the mixture was kept under mechanical stirring overnight. After CHI dissolution, the pH was adjusted to 5.8 with NaOH 1 M to obtain an injectable CNF/CHI suspension biomaterial. In this way, CNF/CHI suspensions were obtained in aqueous conditions. To prepare CNF/CHI suspensions in hydroalcoholic conditions, after CHI dissolution an equivalent weight of 1,2 propanediol as of water (50:50% (*w*/*w*)) was added to the CNF/CHI suspension. The alcohol is not as good a solvent as water for CHI. By adding alcohol, the hydrophobic interactions of the CHI polymer chains are promoted and thereby a higher degree of physical crosslinking should be achieved in the hydrogels.

To obtain CNF/CHI composite hydrogels, the suspensions obtained either in aqueous or hydroalcoholic conditions were neutralized with NaOH 2 M for 1 h using Petri dishes as molds for flat hydrogels. Finally, the hydrogels were washed with distilled water till neutrality, complete removal of the alcohol (hydroalcoholic route) and neutralization salts. CNF/CHI composite hydrogels of around 0.7–2 mm thickness were prepared of different CHI concentrations (1.5%; 1.7%; 2.0%; 3.3% (*w*/*w*)) and CNF contents (0.02%; 0.05%; 0.1%; 0.3%; 0.4%; 0.6% (*w*/*w*)) added in the suspension processing step. To characterize the CNF’s dispersion in the CHI matrix, hydrogel composites were freeze-dried and observed at the scanning electron microscope SEM. Even if the analysis was performed in the freeze-dried state with this technique, it allowed getting insight into the dispersion of the CNFs in the scaffold composites originating from the hydrogel processing. The freeze-dried CNF/CHI scaffolds were carefully fractured and gold sputtered in a Polaron SC 7640 from VG Microtech (East Sussex, UK). Then, SEM observations were performed in an Amray 1810 SEM (Amray Inc., Bedford, MA, USA) at an accelerating voltage of 15 kV.

### 2.4. Shear Rheological Tests on CNF/CHI Suspensions

#### 2.4.1. Couette Geometry

The rheological properties of the CNF/CHI suspensions were studied with a stress-controlled rheometer AR-2000 (TA Instruments, New Castle, DE, USA) equipped with a Couette geometry at 25 °C with gap size of 4 mm. The analysis was performed in triplicate in continuous mode in a shear rate $d\gamma /dt=\dot{\gamma }$ ranging from 5 to 150 s^−1^. The flow diagrams were obtained, namely the plots of the steady-state shear viscosity η vs. $\dot{\gamma }$.

##### Model and Determination of the Rheological Parameters

The flow diagrams (η vs. $\dot{\gamma }$) were analyzed with a three-parameter Cross model [48,49,50]:

(1) $$\eta =\frac{\eta_{0}}{1+{\left(\dot{\gamma \tau }\right)}^{1-n}}$$

The Cross equation yields the Newtonian or zero-shear viscosity η_0_, the flow behavior index *n*, and the relaxation time of polymer chains τ. Our methodology consisted of building flow diagrams in an extended shear rate, combining Couette geometry and capillary rheometry by using an injection setup.

#### 2.4.2. Capillary Rheometry Evaluation of Injectability of the CNF/CHI Suspensions

The study of the injectability of the CNF/CHI formulations is useful to characterize the rheological properties during injection at high shear rates and thereby verify the possibility of manual injection. Together with the Couette geometry, the capillary rheometry should allow building the flow diagrams in an extended shear rate range, to establish rheological criteria for the comparison of Newtonian viscosity and injection ability [50].

**Figure 1:**
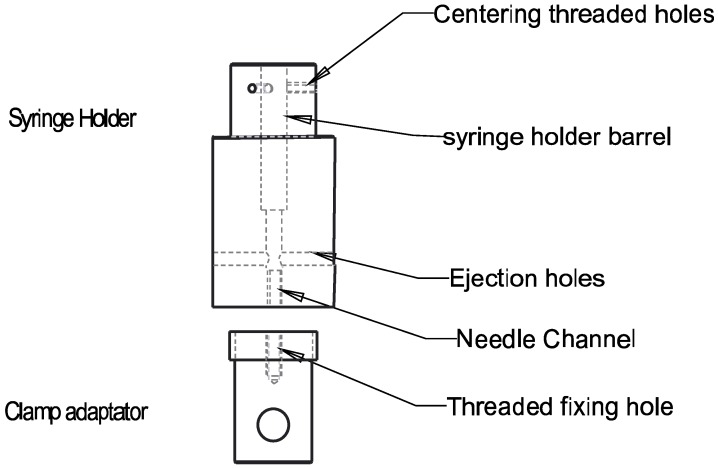
Scheme of the injection setup used for capillary rheology studies with a syringe holder mounted in a tensile/compression testing machine to measure the injectable formulation ejection forces Fe. The syringe body is inserted in the upper part of a barrel holder (10.8 mm diameter hole) and fixed with three polyethylene M4 screws. The needle is centered in a 6.6 mm diameter channel and the extruded formulation can be evacuated by the lateral ducts.

For the injection setup, a syringe holder supporting a 1-mL syringe was integrated into a Criterion tensile testing machine (MTS Systems, Créteil, France) equipped with an upper compression plateau in contact with the plunger of the syringe (Figure 1). This setup is able to mimic a surgeon’s manual injection. The force that has to be applied to the syringe plunger when injecting a formulation depends on the dimensions of the syringe and the needle, injection speed and rheological properties of the injected material. The needle used was a 21G needle (*R* = 0.8 mm, *L* = 80 mm (B. Braun, Melsungen, Germany)). The ejection force was measured using a 500 N force sensor at crosshead speeds (*V*) ranging from 100 to 0.03 mm/min. To report the ejection force *F_e_*, the friction of the empty syringe was subtracted from the ejection force obtained for the syringe containing the CNF/CHI formulation.

##### Determination of the Apparent Viscosity in the Injection Setup

The proposed injection force setup of Figure 1 can be considered a capillary rheometry system where the wall shear stress τ_w_ is mainly due to the shear stress of the formulation flow in the needle. According to the Hagen-Poiseuille law corrected for a non-Newtonian fluid [51], the apparent viscosity η through the syringe and needle is defined as the ratio of the wall shear stress *τ*_w_ and the wall shear rate ${\dot{\gamma }}_{w}$:

(2) $$\eta_{app}=\tau_{w}/{\dot{\gamma }}_{w}$$

where τ_w_ and ${\dot{\gamma }}_{w}$ depend on the needle and syringe dimensions, the crosshead speed *V* and the ejection force determined in the injection setup. According to the force balance, the wall shear stress *τ*_w_ is related to the pressure difference (Δ*P*) along the needle:

(3) $$\tau_{W}=\frac{\Delta P\times R}{2L}$$

with

(4) $$\Delta P=\frac{F_{e}}{\pi \times R_{s}{}^{2}}$$

where *F_e_* is the ejection force, *R_s_* is the internal radius of the syringe, and *R* and *L* are the needle internal radius and length, respectively.

The flow of the formulation in the syringe and needle is considered to be laminar since the Reynolds number *R_e_* = (*R_s_^2^*/*R*)ρ*V*/η (ρ: mass density) is low in the studied shear rate range. For non-Newtonian behavior, the wall shear rate ${\dot{\gamma }}_{w}$ can be expressed [52]:

(5) $${\dot{\gamma }}_{W}=\frac{3n_{wr}+1}{n_{wr}}\times \frac{Q}{\pi \times R^{3}}$$

with

(6) $$Q=V\times \pi \times R_{s}{}^{2}$$

where *Q* is the flow rate, *V* is the crosshead speed and *n_wr_* is the flow behavior index.

By substituting Equations (3) and (5) in Equation (2), the apparent viscosity can be written:

(7) $$\eta_{app}=\frac{F_{e}\times {\left(R/R_{s}\right)}^{4}}{2\times \frac{3n_{wr}+1}{n_{wr}}\times L\times V\times \pi }$$

for which the flow behavior index *n_wr_* can be estimated from the raw data, using the Weissenberg-Rabinowitsch (*wr*) correction in the capillary rheometry (injection setup) [53]. This correction takes into account the wall shear rate ${\dot{\gamma }}_{w}$:

(8) $${\dot{\gamma }}_{W}=\frac{3n_{wr}+1}{4n_{wr}}\times {\dot{\gamma }}_{\mathrm{app}}$$

where ${\dot{\gamma }}_{\mathrm{app}}$ is the apparent shear rate, which can be experimentally determined:

(9) $${\dot{\gamma }}_{\mathrm{app}}=\frac{4Q}{\pi R^{3}}$$

By substituting Equations (8) and (9) in the shear-thinning material model according to the Ostwald-de Waele power law [54], it can be written:

(10) $$\tau_{W}=K\times {\dot{\gamma }}_{w}{}^{n_{wr}}=K\times {\left(\frac{3n_{wr}+1}{4n_{wr}}\right)}^{n_{wr}}\times {\dot{\gamma }}_{app}{}^{n_{wr}}$$

where *K* is the flow consistency index.

Thus, the flow behavior index *n_wr_* can be obtained from the slope of the plot of *Ln*(*τ*_w_) vs. *Ln*(${\dot{\gamma }}_{\mathrm{app}}$). The *n_wr_* value obtained from the Weissenberg-Rabinowitsch correction could be substituted in Equation (7) to determine the apparent viscosity η*_app_*. In the Couette geometry, the flow behavior index was estimated from the fitting of the flow diagrams by using the Cross model of Equation (1) as described above.

### 2.5. Rheological Behavior of the CNF/CHI Composite Hydrogels

Rheological measurements on the CNF/CHI composite hydrogels were performed at 22 °C using a TA Instruments ARES rheometer (New Castle, DE, USA) with 25 mm parallel plates in oscillatory mode. Frequency sweep tests were performed at 0.5% strain with frequency varied from 100 to 0.01 rad/s. The viscoelastic properties (storage modulus G′, loss modulus G″) of the gels were evaluated. The values of G′ and G″ at the plateau for low angular frequencies (ω ≤ 5 × 10^−2^ rad/s) were reported as the equilibrium values G′_e_ and G″_e_, respectively.

### 2.6. X-ray Synchrotron Scattering Analysis of CNF/CHI Formulations

X-ray synchrotron scattering analyses were performed at the microfocus beamline μSpot, BESSY II at the Helmholtz-Zentrum Berlin (Germany) and at the beamline D2AM/BM2 at the European Synchrotron Radiation Facility (ESRF) (Grenoble, France). At Bessy II, data were collected at a wavelength of λ = 1 Å with a setup that allowed simultaneously measuring small- (SAXS) and wide-angle X-ray scattering (WAXS) using a two-dimensional MARCCD detector. At the μSpot, the synchrotron X-ray beam had a diameter of around 10 μm, which passed through the CNF/CHI formulations (suspensions or hydrogels) placed within a holder with Kapton foil windows. At the ESRF, SAXS data were collected at a wavelength of λ = 0.775 Å using a CCD detector (Roper Technologies Inc., Sarasota, FL, USA). Both at Bessy II and at the ESRF, silver behenate was used as standard to calibrate the scattering vector q-range, and transmission corrections and background subtraction were made.

### 2.7. Suitability of the CNF/CHI Sol/Gel Formulations for Application in IVD Tissue Engineering

#### 2.7.1. Cell Culture of Human Fibroblasts on CNF/CHI Hydrogels

To evaluate the suitability of the CNF/CHI gelled formulations for intervertebral disc tissue engineering, experiments were performed with fibroblast cells which were cultured on the CNF/CHI hydrogels. The aim was to determine if cells are negatively impacted by the presence of the CNF in the CHI hydrogel matrix. Human dermal fibroblast (HFIB-D) cells were grown in T75 (75 cm^2^) cell culture flasks (Sarstedt, Nümbrecht, Germany). The cells were cultured in Dulbecco’s modified Eagle’s medium (DMEM(1x) + GlutaMAXTM (Gibco, Thermo Fisher Scientific, Leicestershire, UK) with low glucose, glutamine, sodium pyruvate, supplemented with 10% fetal calf serum (FCS) and penicillin–streptomycin at 37 °C in a humidified atmosphere of 5% CO_2_. Upon 90% confluence, cells were rinsed twice with phosphate buffered saline PBS (Gibco, Thermo Fisher Scientific, Leicestershire, UK) followed by detachment with Trypsin/Ethylenediaminetetraacetic acid (EDTA) for 5 min and neutralization with the corresponding cell culture medium. After detachment, cells were spun down in a centrifuge for 5 min at 1000 rpm. The supernatant was taken off and cells were diluted into the culture medium. CNF/CHI hydrogel pieces were put in 12-well cell culture plates, covering the whole well surface for their subsequent use for cell growth. To this end, 500 μL of suspension of the cells in culture medium as above were added on the hydrogel surface, considering a starting loading of 100,000 cells per well on average. The HFIB-D cells seeded in triplicates on hydrogels of different CHI concentrations and CNF contents were kept at 37 °C in CO_2_ incubation. Cells seeded in empty wells (i.e., without the hydrogel) were used as control.

##### Live/Dead Staining of Cells

The viability of the cells was qualitatively evaluated to get an indication of whether fibroblasts survive in the presence of the CNF in the CHI matrix. Cultivation of at least 6 days was possible and proliferation was observed. The cells were inspected for viability by fluorescent staining with a live/dead staining kit: Calcein AM/ethidium-homodimer-1, LIVE/DEAD™ Viability/Cytotoxicity Kit, (Thermo Fisher Scientific, Leicestershire, UK). After 24 h of HFIB-D cell culture on the composite hydrogels as described above, a cell washing step was performed with Hank’s Balanced Salt Solution (HBSS, Gibco, Thermo Fisher Scientific, Leicestershire, UK) and the freshly prepared Live/Dead solution was added to each sample. Samples were kept at incubation conditions for 15 min. After staining, the wells were imaged using a confocal laser-scanning microscope (Leica TCS SPE, Wetzlar, Germany). As LIVE indicator, Calcein AM marks cell cytoplasm in green fluorescence; and as DEAD indicator, ethidium homodimer-1 stains cell nucleus in red fluorescence.

#### 2.7.2. Cellulose Nanofibers Cytotoxicity Evaluation

Cytotoxicity evaluation of CNFs was assessed accordingly to the method NFEN30993-5 ISO 10993-5 [55,56]. Human dermal fibroblasts Cells (HFIB-D), cultured in Dulbecco’s Modified Eagle’s Medium with Glutamax (Gibco, Thermo Fisher Scientific, Leicestershire, UK) containing 10% fetal calf serum (FSC, Eurobio, Courtaboeuf, France); and human bone marrow stromal cells (HBMSCs), cultured in Alpha MEM Medium without ascorbic acid with Glutamax (Gibco—Life Technologies) containing 10% fetal calf serum (FSC, Eurobio, France), were used for testing 4 concentrations of CNFs suspensions: 100, 50, 25 and 10 µg/mL. The cells were seeded at a density of 15,000 cells/cm^2^ in 96-well microtiter plates (Nunc, Denmark) and the culture was maintained at 37 °C for 48–96 h after cell plating. At subconfluency, the medium was replaced by the different concentrations. Culture medium without material and 0.1% solution of Triton X100 (known to be cytotoxic) were processed under the same conditions to provide negative and positive controls, respectively. The medium was removed and replaced by various concentrations (100, 50, 25 and 10 µg/mL) in the culture medium for 24 h at 37 °C (*n* = 6 for each condition). At the end of the incubation period, cell viability (Neutral red (NR) assay) and cell metabolic activity (MTT assay) tests were performed [57,58].

### 2.8. Injection Experiments in Intervertebral Discs of Animal Models

All used animal and human subjects were carried out according to The French and the National Institutes of Health Guide for the Care and Use of Laboratory Animals by completing the legal forms and having all the required authorizations (legal and ethical). The experimentations on animal subjects were performed by authorized persons in labelled facilities (Institute C. Bourgelat, VetAgro Sup, Lyon Veterinary School, France). The experiments received ethical agreement (Proposal Number 69.127.05.05, Ethical committee agreement number 1440).

#### 2.8.1. MRI and Histological Observations

The localization of formulations injected ex vivo in disc pig models was followed by magnetic resonance imaging (MRI). As the formulation mainly consists of water like in the native biological tissue, a negligible concentration of 4 × 10^−4^ mol/L of gadolinium-based MRI contrast agent (DOTAREM^®^) was incorporated into the CHI-based formulation. It was verified that the mixture was homogeneous and the MRI contrast agent did not affect the physicochemical properties of the formulation (viscosity, gelation). MRI was performed with a MRI E-scan XQ device (Esaote S.p.A, Italy) at 0.2 T [59]. T2-weighted sagittal views were performed of the region of interest in the thoracic-lumbar spine discs before and after formulation injection (see Supplementary Material).

In histological sections of pig model discs, staining with hematoxylin-eosine was performed, including those discs injected in vivo with the formulation (see Supplementary material) [60]. The hematoxylin stains acidic cell compounds, like nuclei DNA. blue/purple. Eosine stains the cytoplasm of the cells pink, and the collagen a range of more or less intense pink, depending on the acidophilic of the different compounds. Eosin stains chitosan pink.

#### 2.8.2. Biomechanical Studies on Discs Injected with CNF/CHI Formulations

The injectability of the CNF/CHI formulations into the disc nucleus was validated in rabbit and pig models. Around 10 μL and 140-205 μL of CNF/CHI suspensions containing 2% (*w*/*w*) CHI and 0.3% (*w*/*w*) CNF were injected ex vivo in lumbar-thoracic disc regions of a white rabbit and a growing pig (4 months, ca. 50 kg weight), respectively. Injections were performed using a 25G needle (0.5 × 16 mm, BD Microlance^TM^ 3, BD, Heidelberg, Germany) connected to a 1-mL syringe (BD Luer-Lok™, BD, Heidelberg, Germany) as shown in Figure S2 in Supplementary Material. Approximately 140 µL were injected in the nucleus of the pig discs L1-T15, T13-T14, T11-T12 and T9-T10, hereon called PI (pre-injected disc). Radiographies of the spine were taken to measure the disc height before and after injections. Then, the different spine motion segments (injected or not), which consist of one disc and half of the two adjacent vertebrae [9], were cut parallel to the corresponding vertebral endplates. To assess the disc biomechanics, the motion segments were tested in compression mode by using a Shimadzu Autograph AG-X plus (Kyoto, Japan). The mechanical test consisted of compressing at a displacement rate of 1 mm/min up to a force of 400 N. Then, the unloading was carried out at the same displacement rate up to 5 N. Five loading/unloading cycles were performed. Afterwards, the motion segment was quickly compressed at a rate of 10 mm/min up to 400 N and at the given displacement (strain) a stress relaxation experiment was followed for 15 min. As previously discussed, water loss and disc height decrease should occur during disc compression. Those discs, which had not been initially injected (L1-L2, T14-T15, T12-T13, T10-T11 and T8-T9 (hereon called S (healthy disc))), were then injected after a first mechanical testing as above. The disc height evolution was measured from the contact difference registered by the upper plateau of the compression equipment, before and after injection of the disc with the CNF/CHI formulation. After the first mechanical testing, injection of up to 205 µL of formulation could be performed. Then, mechanical testing was again performed as above on these injected discs: L1-L2, T14-T15, T12-T13, T10-T11 and T8-T9, hereon called I (injected disc). After test completion, all IVDs were dissected to estimate the disc transverse areas A, whose values were used to calculate the apparent stress σ = F/A.

## 3. Results and Discussion

The methodology followed to prepare the formulations of cellulose nanofiber-filled CHI solutions provided a homogeneous dispersion of the nanofibers in the CHI solution. The obtained suspensions were stable without any apparent CNF macro-aggregates (see Figure S1 in Supplementary Material).

### 3.1. Rheological Properties of CHI/CNF Injectable Formulations

#### 3.1.1. Couette Rheometry

**Figure 2:**
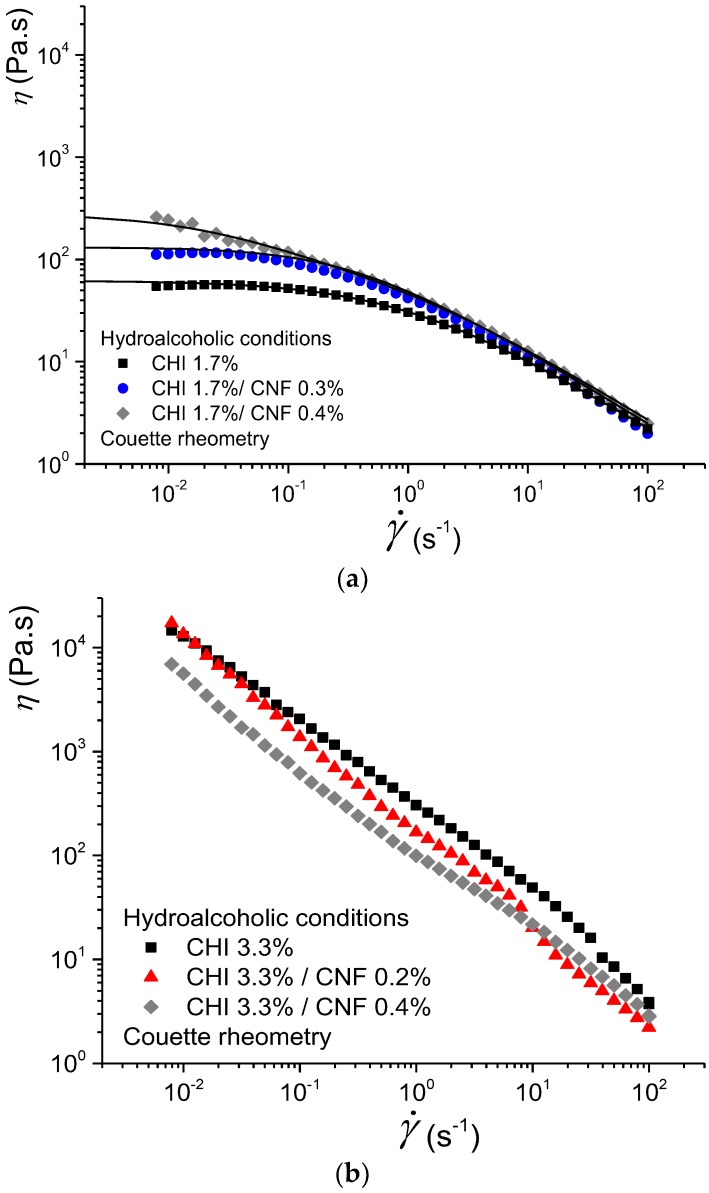
Evolution of viscosity η versus shear rate ${\dot{\gamma }}_{w}$ obtained by Couette geometry for chitosan CHI solutions and cellulose nanofiber-filled chitosan CNF/CHI suspensions of (**a**) 1.7% and (**b**) 3.3% (*w*/*w*) chitosan, containing different CNF contents (0.2–0.4% (*w*/*w*)) and obtained in hydroalcoholic conditions.

Figure 2 displays the flow behaviors of CNF-filled CHI solutions (CNF/CHI suspensions) at different CHI concentrations (1.7% and 3.3% (*w*/*w*)) and different CNF contents obtained in hydroalcoholic conditions. The rheological behavior was strongly affected by the CHI concentration. A flow behavior with Newtonian plateau was clearly observed at low CHI concentration of 1.7% (*w*/*w*), whereas no Newtonian plateau could be observed at high CHI concentration such as 3.3% *w*/*w*. For the latter, the flow behavior was characterized by much higher viscosity values (i.e., above 10^4^ Pa.s at $\dot{\gamma }$ ~ 10^−2^ s^−1^) and a decrease of the apparent viscosity occurred in the whole shear rate range (Figure 2). The evolution of η with $\dot{\gamma }$ was approximately η ~ 1/$\dot{\gamma }$, which is a signature of a gel state. Thus, for a CHI of high molecular weight (*M*_w_ = 6.11 × 10^5^ g/mol) and low degree of acetylation (DA = 2.5%) as used in this work, formulations with CHI concentration ≥3.3% (*w*/*w*) display a rheological behavior close to a soft hydrogel with limited injectability with standard injection conditions.

#### 3.1.2. Building of Flow Diagrams in an Extended Shear Rate Range. Couette Geometry and Capillary Rheometry from the Injection Experiments

**Figure 3:**
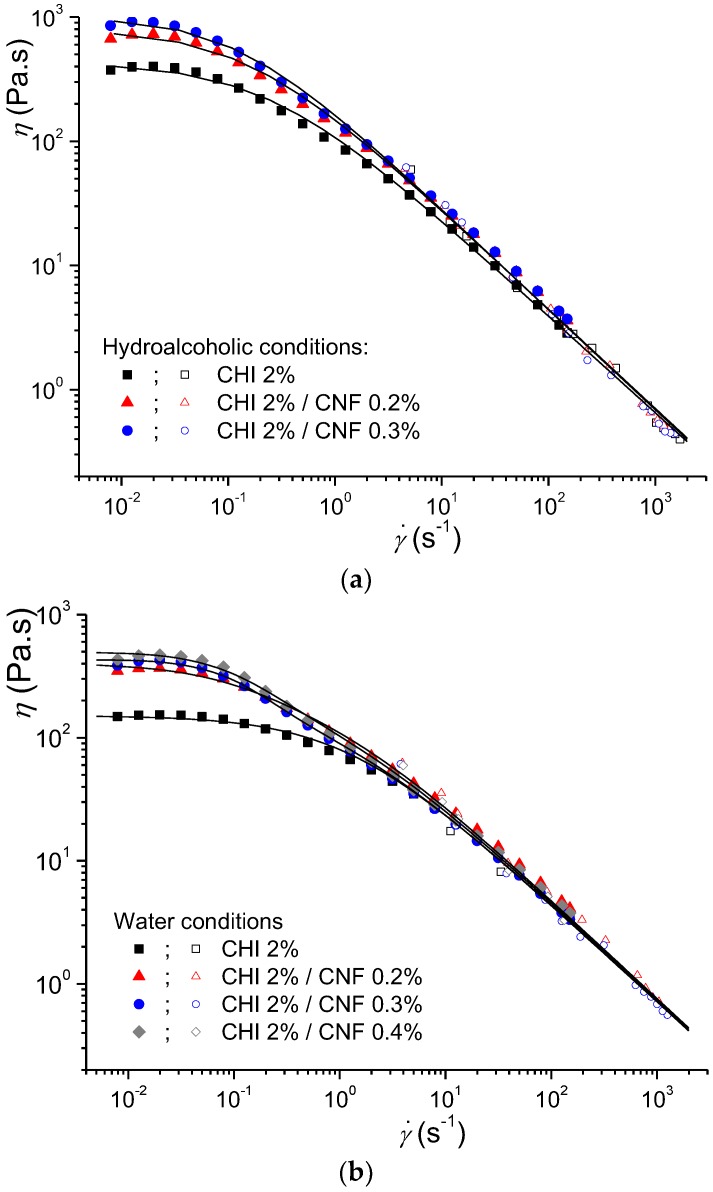
Flow diagrams and their modeling in an extended shear rate range, including Couette and capillary rheometry using the injection setup, of 2% (*w*/*w*) CHI solutions and CNF/CHI suspensions with 2% (*w*/*w*) CHI and different CNF contents, obtained in different solvent conditions: (**a**) hydroalcoholic and (**b**) aqueous. The solid lines display the modeling with the Cross law (Equation (1)) for the CHI solutions and with a “double” Cross law (Equation (11)) for the CNF/CHI suspensions.

We performed a more detailed study at an intermediate CHI concentration (2% (*w*/*w*)) with CNF contents from 0.2 to 0.4% (*w*/*w*) and for the different solvent conditions: hydroalcoholic and aqueous. We studied the rheological behavior in an extended range of shear rates by using both the Couette geometry and the capillary rheometry using the above-described injection setup [50]. Comparing the injection of different formulations with the same syringe type and the same ejection speed, a difference in the extrusion force may be interpreted as a difference in the viscosity of the formulation to be injected. Thus, for the development of an injectable biomaterial for a given application, the combined characteristics of the syringe/needle and the rheological behavior of the injectate should be adjusted to allow an injection force value below 15 N for safe and easy injection procedures. The CNF/CHI formulations were easily injected using standard needles. The ejection force *F_e_* analysis was performed using a 1-mL syringe (*R*_s_ = 2.55 mm (B. Braun) and a 21G needle (*R* = 0.8 mm, *L* = 80 mm (B. Braun, Germany)), for which the obtained *F_e_* values were smaller than the typical threshold of 15 N. The formulations evaluated in this study corresponded to the requirements established for injectable systems using a standard syringe and needle for medical practices. It demonstrated the suitability of these formulations in medical applications. Table 1 shows the flow behavior index *n*_wr_ values obtained from the slope of the plot of *Ln*(*τ_w_*) vs. *Ln*(${\dot{\gamma }}_{\mathrm{app}}$) (Equation (10)) for different CNF/CHI formulations. To calculate η*_app_*, the ejection force *F_e_* and *n*_wr_ values obtained at crosshead speeds *V* varying from 0.03 to 100 mm/min were substituted in Equation (7). Figure 3 (empty symbols) displays the apparent viscosity η*_app_* obtained at high shear rate by capillary rheometry using the injection setup.

Figure 3 shows the flow diagrams in an extended shear rate range of more than 5 decades, by considering both Couette geometry and capillary rheometry from the injection experiments. The obtained results are comparable to the shear-thinning behavior of solutions only containing chitosan, i.e., without CNFs [50,61,62,63]. The good correspondence of the two set of experiments (Couette and capillary rheometry) allowed simultaneous analysis of the rheological behaviors. The extended flow diagram of the “pure” chitosan solutions could be modeled with the Cross law (Equation (1)). The obtained Newtonian viscosity η_0,*Chi*_, the relaxation time τ_Chi_, and the exponent *p_Chi_* = 1 *− n_Chi_* (*n_Chi_*: flow behavior index) for pure CHI solutions are reported in Table 2. The CNF/CHI suspensions exhibited more complex flow diagrams (Figure 3), with higher Newtonian viscosities measured in the low shear rate range (*dγ*/*dt* < 1 s^−1^) and shear thinning occurring in two different regimes. These two-step flow diagrams could be modeled with a double Cross law:

(11) $$\eta =s\frac{\eta_{0,Chi}}{1+{\left(\dot{\gamma }\tau_{Chi}\right)}^{p_{Chi}}}+\frac{\eta_{0,CNF}}{1+{\left(\dot{\gamma }\tau_{CNF}\right)}^{p_{CNF}}}$$

where η_0,*CNF*_, *τ_CNF_*, and *p*_CNF_ = 1 − *n_CNF_* are the flow parameters of CHI chains, possibly due to chains interacting with CNFs in the CNF/CHI suspensions in the slower flow regime. Fitting used a Levenberg-Marquardt nonlinear regression algorithm in Octave 4.4.0 programming environment [50]. Finally, the flow data of the CNF/CHI suspensions with CHI concentration of 1.7% (Figure 2) and 2% (*w*/*w*) (Figure 3) could be modeled with the double Cross law (Equation (11)). For the CHI solutions, Table 2 shows the flow parameters obtained for the CNF/CHI suspensions.

Summarizing, at CHI concentrations around 2% (*w*/*w*), both CHI solutions and CNF/CHI suspensions exhibited higher viscosities at low shear rates. The presence of CNF increased the Newtonian viscosity measured at low shear rates. With increasing shear rate, viscosity similarly decreased in both CHI solutions and CNF/CHI suspensions, revealing a shear-thinning behavior with power law (η ~ 1/${\dot{\gamma }}^{1-n}$) (Figure 3).

The viscosities of the formulations obtained in hydroalcoholic media were higher than in aqueous conditions. In pure CHI systems, this was previously explained by the possibility of chitosan inter-chain crosslinking involving the diol and hydrogen bonds formation as demonstrated by means of rheological experiments in dynamic conditions [64]. Table 2 shows higher relaxation times obtained in the hydroalcoholic conditions; that is, reduced CHI chain mobility in comparison to aqueous conditions [61,62]. In the CNF/CHI formulations two different chain relaxation phenomena were displayed (Figure 3). In the CHI reference solutions, the main chain relaxation, being dominant at high shear rates and corresponding to the disentanglement of the chain transient network, showed relaxation time of 0.8 s in aqueous solutions with 2% (*w*/*w*) CHI, and closer to 1 and 4 s in hydroalcoholic solutions with 1.7% and 2% (*w*/*w*) CHI, respectively (Table 2). At low shear rates, in the presence of CNFs, a second relaxation occurs with relaxation times in the order of 10 s (independently of the CNF content).

### 3.2. Viscoelastic Properties of the CNF/CHI Hydrogels

**Figure 4:**
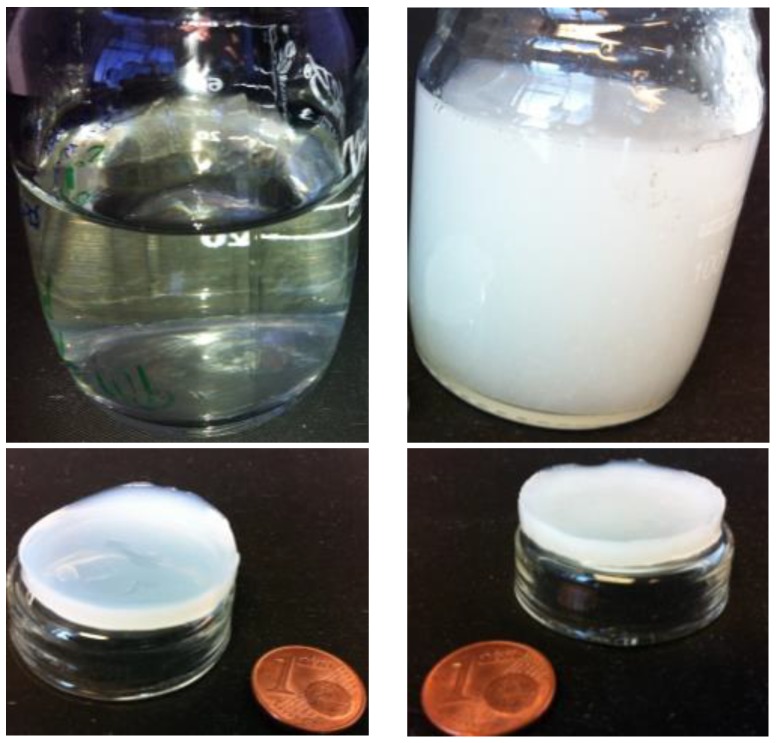
Reference solution (*Top-left*) and hydrogel (*Bottom-left*) of chitosan 2% (*w*/*w*). Solution of CHI 2% (*w*/*w*) filled with cellulose nanofibers 0.4% (*w*/*w*) (CNF/CHI suspension, *Top-right*) and resulting CNF/CHI composite hydrogel (*Bottom-right*). All samples were obtained by the aqueous route.

Figure 4 shows examples of CHI and CNF/CHI composite hydrogels obtained after neutralization of the corresponding injectable formulations. Also in the hydrogels, a homogeneous dispersion of the CNFs in the CHI matrix was obtained (see also Figure S1 in Supplementary Material).

**Figure 5:**
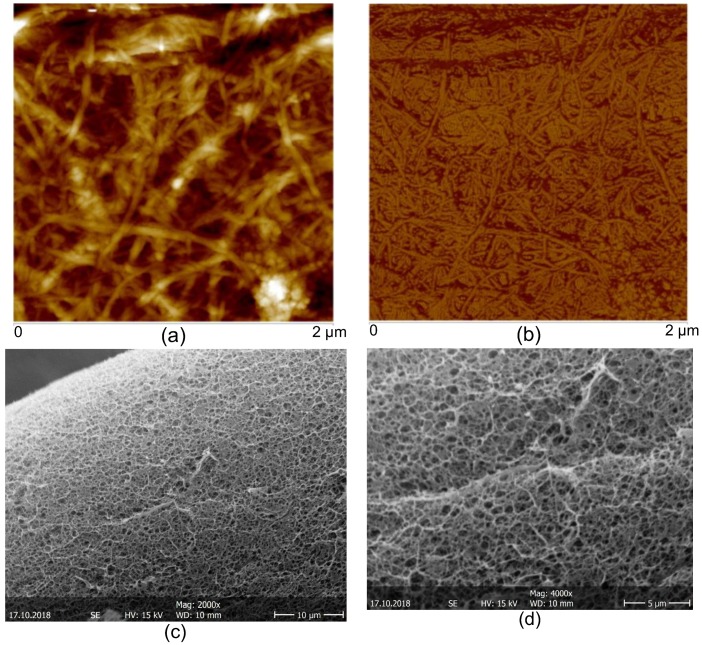
Atomic force microscopy AFM topography (**a**) and phase (**b**) images of the cellulose nanofibers CNFs used to reinforced the cellulose nanofiber-filled chitosan CNF/CHI hydrogel composites. SEM micrographs (**c**,**d**) of scaffolds obtained after freeze-drying of the CNF/CHI hydrogels (CHI 2%/CNF 0.3%).

The AFM images of the CNFs dried on mica substrate revealed an entangled network of interconnected nanofibrils with average width of 35.2 ± 8.1 nm and bundles up to 100 nm width (Figure 5). The relative larger width of the fibrils observed by this method could be due to the drying of the fibrils inducing the formation of aggregates, as previously reported by Pääkkö et al. [47]. As mentioned above, in this work the preparation of the CNFs was performed following a methodology similar to that used by Pääkkö et al. [47], in which a mechano-enzymatic hydrolysis leaves long nanoscale fibrils preserving the native cellulose I crystalline allomorph with partly amorphous regions, which nanofibrils are able to inherently entangle. The preserved native cellulose I allomorph and the intramolecular hydrogen bonding in the obtained nanofibrils lead to improved mechanical properties noted higher than for fibrils consisting of regenerated cellulose II allomorph. Such long entangled nanofibers as those produced here should be feasible as percolating nanoreinforcement of hydrogel composites. The SEM micrographs of the CNF/CHI hydrogels which were freeze-dried for composite observation revealed a sponge-like network microstructure with interconnected pores. In all studied formulations, a regular network structure with non-distinguishable cellulose nanofibers was observed, which can be due to the good interfacial compatibility of the polysaccharide nanofibers and matrix allowing for good dispersion of the CNFs (Figure 5).

**Figure 6:**
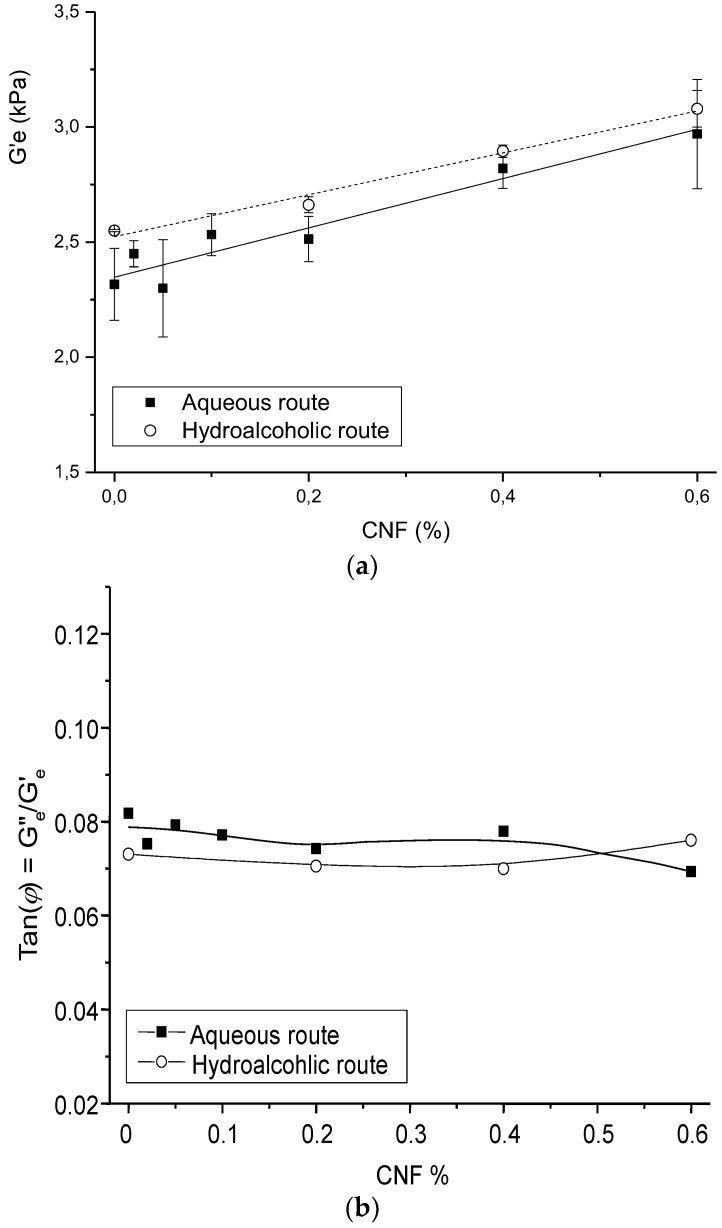
Evolution of the equilibrium storage modulus G′e (**a**) and dissipation ratio (Tan(*φ*) = Ge″/Ge′) (**b**) with the increase of the CNF content in the CNF/CHI hydrogel composites, obtained by the aqueous and hydroalcoholic routes.

Figure 6 shows the impact of CNF content on the hydrogel equilibrium storage modulus G′_e_ and dissipation ratio Tan(φ) = G_e_″/G_e_′. The evolution of G′_e_ was analyzed through the generalized Einstein relation:

(12) $${G^{\prime }}_{e}={G^{\prime }}_{e,m}\cdot \left(1+k_{E}\cdot f_{V,CNF}\right)$$

where G′_e,m_ is the modulus of the hydrogel matrix, *f_V_*_,CNF_ is the volume fraction of CNF, and *k*_E_ is the Einstein factor (2.5 for spherical particles; 2L/D for elongated particles/fibers of length *L* and diameter *D*). As expected, the *G*′_e,m_ value for CHI hydrogels obtained by the hydroalcoholic route was higher than the modulus of hydrogels prepared by the aqueous route (2.52 kPa and 2.35 kPa, respectively). The global Einstein factor obtained for all CNF/CHI hydrogel composites was higher and close to 50, revealing the reinforcement effect of the cellulose nanofibers.

**Figure 7:**
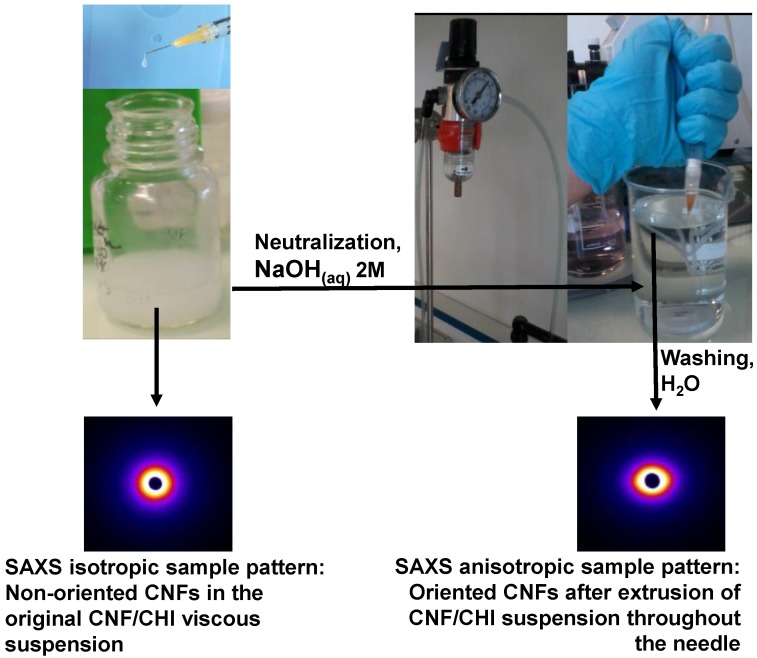
X-ray scattering analyses of the starting CNF/CHI formulation, whose small angle X-ray scattering SAXS pattern corresponds to isotropic behavior; and of the formulation after extrusion through the needle and neutralization, whose SAXS pattern corresponds to anisotropic behavior, revealing the orientation of the CNFs.

Summarizing, the higher Newtonian viscosity measured at low shear rates in the CHI solutions filled with CNFs, in comparison to the CHI reference solutions, and their shear-thinning behaviors with similar viscosities and flow exponents at high shear rates, could be explained due to the CHI polymer chain relaxation (chain disentanglements) impacted by the CHI concentration and molecular weight [65], rather more than by the presence of the nanofibers. Nevertheless, the amplitude of the CHI chain relaxation slightly increases with the CNF content. This low shear rate relaxation could thus be ascribed to chains with slower dynamics, interacting with CNFs. As mentioned above, the surface of CNFs used in this work is weakly charged with carboxylate moieties displaying a surface charge density of 40–80 mmol/kg [66,67]. Weak electrostatic interactions could be established between the CHI polycation and the CNF polyanion, allowing for stress transfer from the CHI matrix to the nanofibers. In the CNF/CHI suspensions, the establishment of a rigid cellulose network with permanent CNF-CNF interactions should not lead to the observed flow behavior, since a gel-like behavior with η ~ 1/$\dot{\gamma }$ should then be observed. Instead, CHI chains should absorb on the surface of CNF and play a role in the bridging of nanofibers [68,69], and also result in formation of entanglement between the adsorbed chains and the other chains in the solution. Introduction of CNF, even at low concentration, is likely to impact the dynamics of CHI chains since the surface area of the nanofibers is large [44]. In solution, such dynamic interactions strongly occur in hydroalcoholic solvent. Indeed, in the low shear rate range the value of the CNF-dependent viscosity plateau is higher for hydroalcoholic suspensions (η_0,*CNF*_ in Table 2). It evidences that the interactions between CHI and CNF may be promoted by the presence of 1,2 propanediol [64]. In the context of CNF/CHI suspensions, 1,2 propanediol could help to establish H-bonding between CHI chains and CNF surface and favor hydrophobic interactions between CHI and cellulose. The flow exponent index *n_wr_* values obtained from the capillary rheometry using the injection setup can represent the strain rate sensitivity of the disruption of various types of intermolecular interactions in the formulation, mainly related to CHI polymer. Table 1 shows that the *n_wr_* values were similar for all samples (Table 1). Thus, the suspension flow at high shear rates is dominated by the disentanglement of CHI chains and is not affected by the presence of the CNFs, which should get oriented in the flow direction (Figure 7) [70]. Such orientation was confirmed by comparing the X-ray scattering analysis of the starting CNF/CHI formulation, whose SAXS pattern corresponded to isotropic behavior, with that of the formulation after extrusion through the needle, whose SAXS pattern corresponded to anisotropic behavior revealing the alignment of the CNFs (Figure 7). The similar viscosities of CHI solution and CHI/CNF suspensions observed at the highest shear rates, also studied by capillary rheometry using the injection setup, could be explained by the disruption of the CNF network and orientation of the fibrils to minimize flow resistance.

The CNF/CHI suspensions are envisioned as injectable implants. Thus, the viscosity at high shear rates (injectable conditions) needs to be as low as possible, whereas the viscoelastic properties of the implant at rest should be high enough to ensure a filling effect and localization of the formulation in the vicinity of the injection site. Such a trade-off led, for example, to the definition of rheological criteria able to qualify the performance of dermal fillers, by considering the ratio of Newtonian viscosity to flow viscosity at $\dot{\gamma }$ = 10^4^ s^−1^ [50]. In the present work, the dispersion of CNFs in the CHI solutions increases the ratio of the Newtonian viscosity in comparison to pure CHI solutions, and hence the performance of the filler. By increasing the Newtonian viscosity with the CNFs, we can tune the mechanical properties and resorption kinetics of the implant. Increasing the viscosity fastens the gelation, which may further contribute to avoid leakage of the injectate when the needle is withdrawn. This undesired effect was observed, for example, with the injection of alginate and hyaluronic acid-based solutions [71]. Additional increase of viscoelastic properties could be obtained by the gelation of the formulation, for example, in contact with body fluids, and specially with the addition of the cellulose nanoreinforcement (Figure 6). Finally, the study of the viscoelastic properties of the CNF/CHI hydrogels, resulting from the gelation of the CNF/CHI suspensions, revealed the strong intrinsic reinforcing ability of the CNF nanofibers (with Einstein coefficient *k*_E_ ~ 50), also confirming a good interaction between the nanofibers and the CHI matrix in the hydrogel.

### 3.3. Assessment of the Suitability of the CNF/CHI Formulation for IVD Tissue Engineering

Previously, we performed studies devoted to the ex and in vivo evaluation of CHI-based solutions for the treatment of IVD degeneration [60]. Injection tests were performed in the nucleus pulposus of IVD in a pig model. The MRI image in Figure S3 (Supplementary Material) shows the localization of the product after ex vivo injection in the L6-L7 lumbar disc of 100 μL of a mixture of the CHI solution with the gadolinium-based MRI contrast agent. The injectate could be clearly visualized within the NP in the images (obtained 1 h after injection) and stayed at the implantation site event after physiological mechanical loading of the spine in compression, torsion and bending.

It is also important to understand how the injectate integrates in the nucleus. In the long term, resorption of the initially formed gel and formation of polyelectrolyte complexes between CHI and the NP components are expected. The histological analysis at Day 95 of discs injected in vivo with CHI-based formulation exhibited an architecture resembling that of the healthy disc (see Figure S4 of Supplementary Material) [60]. The disc was clearly distinguished between the cartilaginous end-plates attached to the surface of the two adjacent vertebral bodies (VBs). Within the disc, NP and AF were distinctly observed. Moreover, at Day 95 no calcification of the discs was observed. Discs injected with CHI-based formulations of different CHI concentrations around 2% and 3% (*w*/*w*) showed comparable results with no differences in local tolerance. At a cellular scale, the NP was characterized by the presence of small chondrocyte-like round cells organized in clusters surrounded by an ECM rich in type II collagen and PGs. As expected, the annulus preserved its architecture with concentric fibrocartilage lamellae where the cells were rather fibroblast-like. At Day 1, discs injected with the CHI formulations of Cp = 2% and Cp = 3% showed an average disc height increase of 17 ± 5%. A detailed morphometric analysis at Day 95 showed a mean height of 6.3 mm ± 0.15 mm between the growth cartilages attached to the adjacent vertebrae, an IVD mean height of 3 mm ± 0.05 mm, an IVD mean width of 14 mm ± 0.50 mm; with NP average width of 8.6 mm ± 0.35 mm, NP average sectional area of 19 mm^2^ ± 0.70 mm^2^ and AF average sectional area of 13 mm^2^ ± 1.0 mm^2^. These results are similar to those obtained for the starting healthy disc without injection. This suggests that the initial disc height increases immediately after injection of the CHI-based formulation, and then equilibrates over the long term as a result of the physiological loading of the spine. Ideally, the injectate must withstand significant compression (between 0.1 and 2.3 MPa) without complete dehydration or leakage through the injection channel, and should rehydrate at rest to restore the equilibrium disc height. In addition, the slow in vivo biodegradation, reported for highly deacetylated chitosans (as used in this work: CHI DA = 2.5%) and for crystalline CNFs, should guarantee the long-term support during tissue regeneration to be provided by the CNF/CHI hydrogel composites [72].

On one hand, these observations of the pig model validated the biocompatibility of CHI-based solutions and their in situ-formed hydrogels in the context of viscosupplementation of healthy discs. On other hand, we showed that the rheological behavior determining the injection conditions (at high shear rates) was mainly determined by the CHI solution. Nevertheless, the biocompatibility of the CNFs dispersed within the CHI solutions and hydrogels also should be tested to validate the use of CNF/CHI formulations for IVD tissue engineering applications.

#### 3.3.1. Cell Culture of Fibroblasts on the CNF/CHI Hydrogels

**Figure 8:**
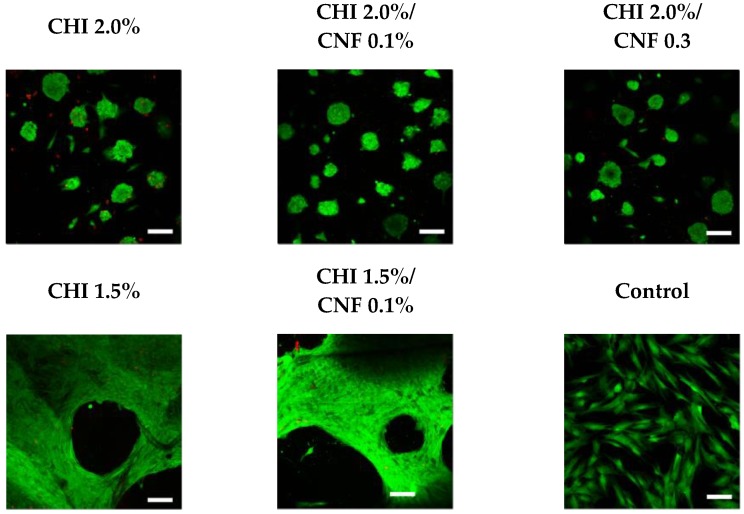
Live-dead assay performed after 24 h of cell culture of human dermal fibroblast (HFIB-D) cells on cellulose nanofiber-filled chitosan (CNF/CHI) hydrogels of different CHI concentrations and CNF contents. Scale bar: 100 μm.

**Figure 9:**
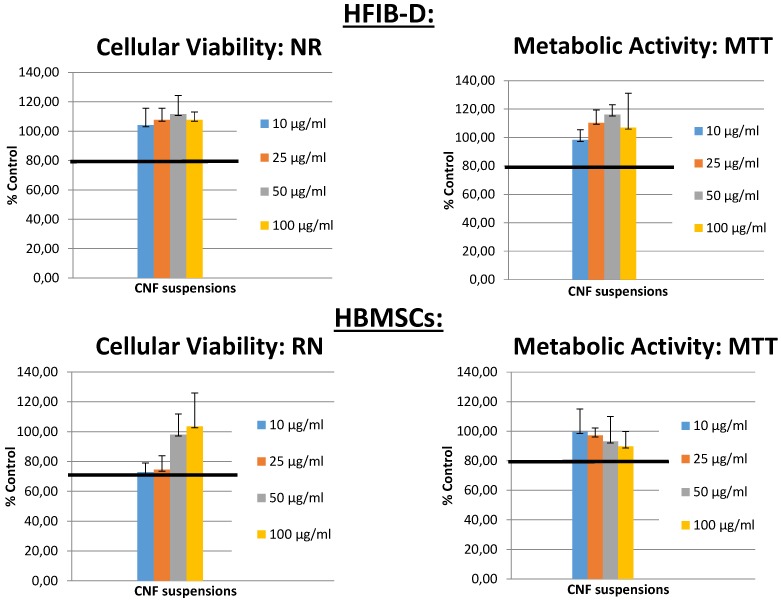
Cytotoxicity evaluation of cellulose nanofibers suspensions showing the cell viability (Neutral red (NR) assay) and cell metabolic activity (MTT assay) for human dermal fibroblasts (HFIB-D) and human bone marrow stromal cells (HBMSCs) for different CNF concentrations.

The biocompatibility of CNF-filled CHI hydrogels with different CNF contents and CHI concentrations was studied in vitro in the cell culture of skin human fibroblasts. Figure 8 shows exemplary results of confocal microscope imaging of Life/Dead assays performed on human dermal fibroblast (HFIB-D) cells, which were cultured on the CNF/CHI hydrogels. The cell viability observed on the CNF/CHI composite hydrogels was similar to that observed on the corresponding “pure” CHI hydrogel reference (Figure 8). For hydrogels prepared with formulations of 1.5% (*w*/*w*) CHI and this CHI concentration combined with 0.1% (*w*/*w*) CNF (i.e., CHI:CNF weight ratio of 15:1), after 24 h practically confluent cell spreading was achieved on both the neat CHI hydrogels and the CHI/CNF composites. For a higher CHI concentration of 2.0% (*w*/*w*), after 24 h the cells still mainly remained in clusters in both the neat CHI hydrogel and the composites of 2.0% (*w*/*w*) CHI with 0.1% (*w*/*w*) CNF (CHI:CNF weight ratio of 20:1), and of 2.0% (*w*/*w*) CHI with 0.3% (*w*/*w*) CNF (CHI:CNF weight ratio of 6.7:1). All studies were performed using hydrogel materials placed at the bottom of the culture wells. On the materials having higher CHI concentrations, such as 2.0% (*w*/*w*), a very good viability was observed even if after 24 h still a formation of cellular aggregates on the surface of the gels was revealed. Then, for cultivation times longer than 48 h a representative spreading was observed. It would indeed be necessary to make more observations at longer times. The cellular behavior differences on the various CHI concentrations could be explained due to the increase of stiffness and density (porosity decrease) of the hydrogel when increasing the CHI concentration, with less accessibility for the cells and reduced adhesion. Materials with lower CHI concentration should allow the formation of a hydrogel network of bigger pore size, approaching a 3D culture environment more relevant for cell growth and adhesion. However, in the proposed biomaterials, interactions between cells by means of junctional proteins could be promoted as have been previously reported [73]. The similarity of the results obtained for CHI alone and for the corresponding CNF/CHI composites suggests that the addition of CNFs does not compromise the biocompatibility of chitosan. As a nanomaterial, CNFs provide a large surface in contact with the CHI hydrogel matrix. This latter probably serves as coating for the cellulose fibril surface, which seems to be favorable for cell spreading and growing. Then, we also evaluated the cytotoxicity of the cellulose nanofibers (Figure 9).

Figure 9 shows the cell viability (Neutral red (NR) assay) and cell metabolic activity (MTT assay) obtained for human dermal fibroblasts (HFIB-D) and human bone marrow stromal cells (HBMSCs) for different CNF concentrations considered in the CNFs cytotoxicity evaluation. The intensity of the obtained colors (red and blue, respectively) was directly proportional to the viability and metabolic activity of the cell population and inversely proportional to the toxicity of the material. Indirect cytotoxicity tests were duplicated for each cement composition. The mean values of absorbance measurements obtained from colorimetric tests and their corresponding standard deviation (±SD) were calculated. Figure 9 shows the results expressed as a percentage of the negative control (plastic) tested in the same experiments. More than 80% of cell viability was observed with a very good metabolic activity of the different investigated cells, demonstrating the good biocompatibility of the CNFs.

#### 3.3.2. Application of the CNF/CHI Formulations to Restore the Disc Height Loss and Viscoelastic Properties

**Figure 10:**
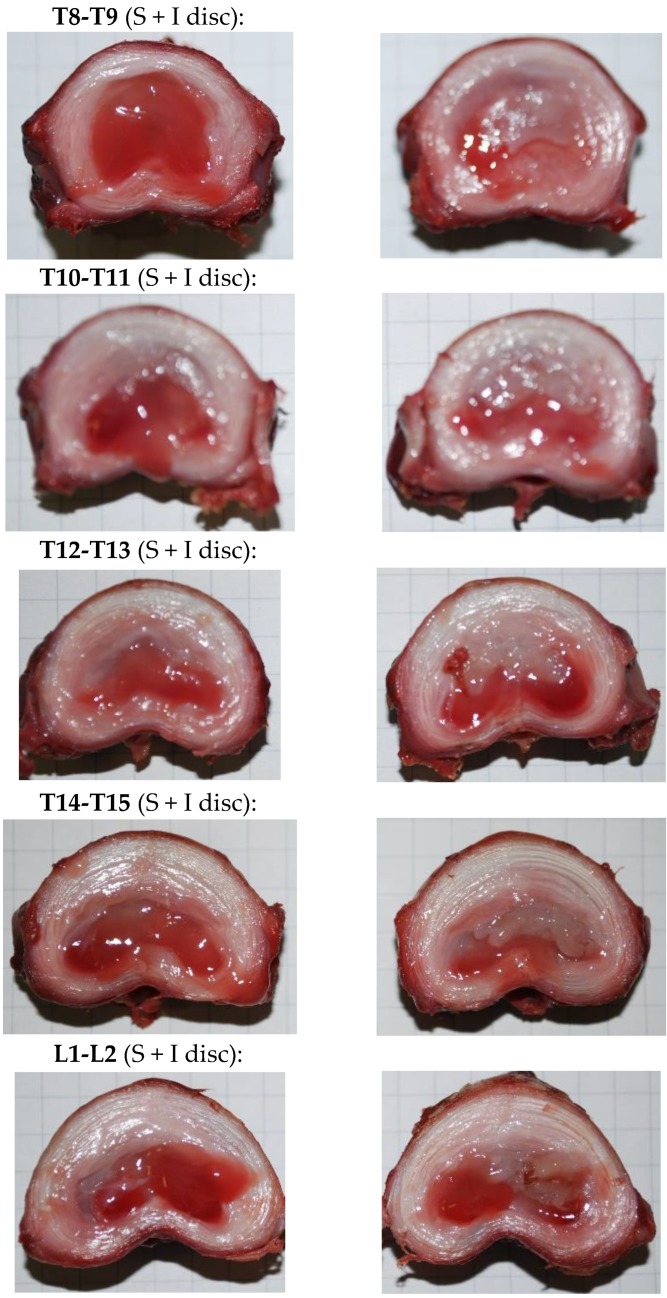
Each row shows the corresponding two transverse cut pieces produced from the dissection of different thoracic-lumbar discs of a pig model, after nucleosupplementation with injection of CNF/CHI formulation (2% (*w*/*w*) CHI/ 0.3% (*w*/*w*) CNFs). After injection and before discal dissection, the spine motion segments were subjected to five compression loading/unloading cycles and stress relaxation experiment as described above.

Again, the injection ex vivo of CNF/CHI formulations was easy with thin 25G needles (0.5 × 16 mm, BD Microlance^TM^ 3, BD, Heidelberg, Germany) and the product did not leak after injection. This latter was further checked after the spine motion segments of injected discs were subjected to compression. The injectates remained located inside the nucleus and were found as gels integrated within the original nucleus, as observed after discal dissection. Figure 10 shows images of dissected discs that were injected and subsequently subjected to the compression loading/unloading cycles as described above. In the images, the gelled formulation appears on the top-right part of the NP corresponding to the position where the formulation was injected by the veterinary surgeon.

**Figure 11:**
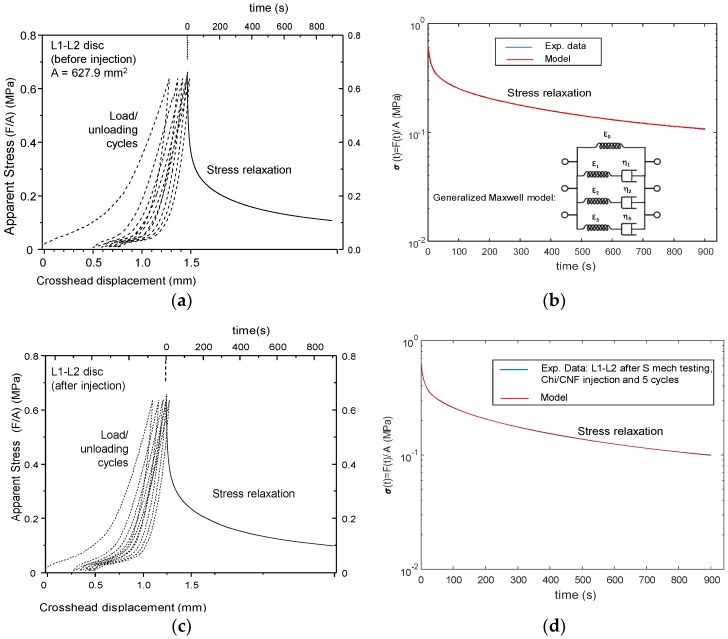
Curves of mechanical tests performed ex vivo in intervertebral disc IVD pig model. Non-injected disc L1-L2 (S): (**a**) Five compression loading/unloading cycles performed at 1 mm/min up to a maximal load of 400 N and, then, stress relaxation for 15 min; (**b**) Modeling of the obtained stress relaxation experiment by using a generalized Maxwell model. After mechanical testing, after injection with 205 µL of cellulose nanofiber-filled chitosan CNF/CHI formulation (2% (*w*/*w*) CHI/ 0.3% (*w*/*w*) CNFs) of the same disc L1-L2 (I), this latter was subjected again to mechanical testing (**c**): Five compression loading/unloading cycles performed at 1 mm/min up to a maximal load of 400 N and, then, stress relaxation for 15 min; (**d**) Modeling of the obtained stress relaxation experiment by using a generalized Maxwell model.

**Figure 12:**
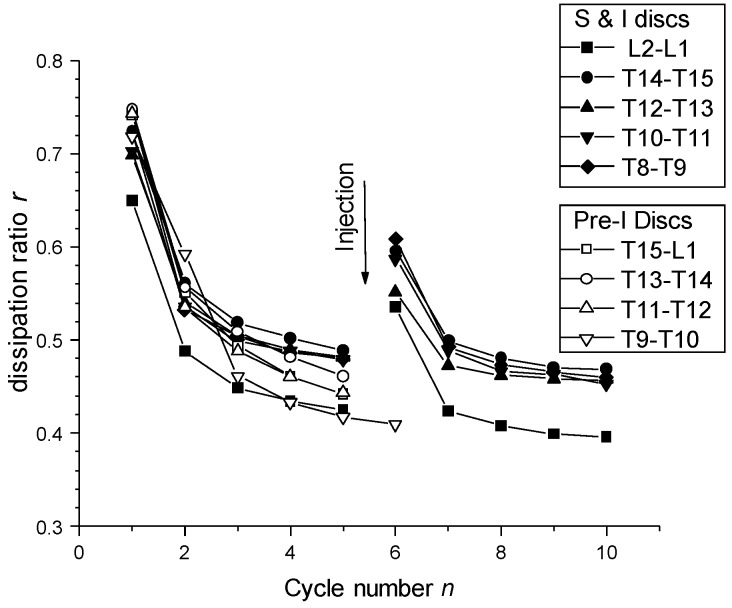
Mechanical dissipation ratio of the compression loading/unloading cycle analyses of healthy and injected discs (S and I); and discs pre-injected (Pre-I) before mechanical testing (see Section 2. Materials and Methods). Analyses were performed in different thoracic-lumbar (T-L) discs of a pig model. Injection of CNF/CHI formulation induced rehydration of the disc and thereby a partial recovery of the mechanical dissipation ratio.

**Figure 13:**
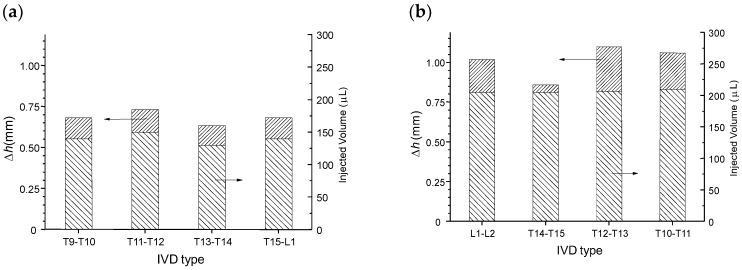
Increase of IVD height after intradiscal injection of cellulose nanofiber-filled chitosan CNF/CHI formulation (2% (*w*/*w*) CHI/ 0.3% (*w*/*w*) CNFs). (**a**) Healthy discs (S); (**b**) discs injected (I) after five compression loading/unloading cycles up to 400 N load and a stress relaxation experiment performed for 15 min (see Section 2. Materials and Methods).

The impact of injection could be evaluated from the evolution of disc height and mechanical behavior (Figure 11, Figure 12 and Figure 13). The first compression loading/unloading cycle corresponded to the largest mechanical dissipation hysteresis. The successive cycles were less dissipative and shifted to higher crosshead displacements. The disc height decreased after each cycle. The equilibrium cycle was reached close to the fifth cycle. Then, the stress relaxation experiment was conducted for 15 min (continuous lines in Figure 11). Such relaxation was not a simple mono exponential decay and instead revealed three relaxation modes which could be modeled according to the generalized Maxwell model:

(13) $$\sigma \left(t\right)=\sigma_{0}+\sigma_{01\cdot }e^{-t/\tau 1}+\sigma_{02\cdot }e^{-t/\tau 2}+\sigma_{03\cdot }e^{-t/\tau 3}$$

where σ_0_ is the unrelaxed stress, and σ_01_, σ_02_ and σ_03_ are the relaxation amplitudes of the three relaxation modes with relaxation times τ_1_, τ_2_ and τ_3_, respectively. Thus, the stress at zero time is Σσ_i_ = σ_0_ + σ_01_ + σ_02_ + σ_03_; the relaxable stress fraction can be defined as the ratio (σ_01_ + σ_02_ + σ_03_)/Σσ_i_ and the (non-relaxable) elastic fraction is defined as the ratio σ_0_/Σσ_i_. As shown in Figure 9, such modeling reproduced the experimental data very well. Table 3 shows the modeling parameters of experiments performed on different discs of the thoracic-lumbar (T-L) region of a pig model, where S corresponds to non-injected healthy discs; PI to pre-injected discs; and I to injected discs whose injection was performed after the first mechanical testing consisting of five compression load/unloading cycles followed by a stress relaxation experiment as explained above.

The values of relaxation times systematically exhibited a slow mode (τ_1_ ~ 300–600 s), a fast mode (τ_3_ ~ 5–8 s) and an intermediate relaxation mode (τ_2_ ~ 50–100 s). The injection (I) or pre-injection (PI) did not significantly change these relaxation times and the repartition of these relaxation modes. Figure 12 displays the evolution of the dissipation ratio, i.e., the ratio of the area located between loading and unloading curves normalized by the area under the loading curve. It represents the fraction of dissipated mechanical energy during one cycle. Filling the disc allowed restoring, in a relatively good extension, the initial hysteresis measured on the first cycles of a series.

Figure 13a displays the injection volumes and corresponding disc height increases obtained for healthy discs, just after preparation of the motion segments (vertebra + disc + vertebra). Figure 13b displays the injection volumes and height increases obtained for samples injected after the previous mechanical test (five compression loading/unloading cycles followed by one stress relaxation experiment).

The average intradiscal injectable volume increased from 140 μL in healthy discs to 205 μL in compression/decompression tested discs. Thus, after mechanical testing a decrease of the disc height was induced (close to 0.8 mm according to Figure 13), associated with a decrease of IVD hydration, allowing for injection of more volume of the formulation (65 μL). Such post-mechanical disc height loss was compensated by injection of the formulation, which re-established the disc height (see Figure 13b). Such injection also rejuvenated the mechanical properties close to that of the healthy disc behavior (Figure 12). Indeed, a large first hysteresis on the first cycle could be observed, followed by dehydration and cycle shifting with stabilization. Thus, mechanical testing induced disc dehydration during cycling and relaxation. This dehydration could be compensated by intradiscal injection, i.e., nucleosupplementation, so that the disc height and mechanical properties again approached that of the healthy disc.

Summarizing, injection of CNF/CHI formulation in the disc nucleus of healthy discs neither altered their mechanical properties nor their dissipation behavior during cyclic mechanical loading (Figure 11 and Figure 12). Strong loads on spine motion segments (F = 400 N, ~weight of the animal) induces a significant decrease of IVD height (Δ*h* ~ 0.8 mm) related to disc dehydration, which significantly occurs in the nucleus. Injection and rehydration in discs which were exposed to mechanical cycling loading, as occurring in daily activities, could restore both disc height and mechanical behavior approaching that of the original healthy disc, with dissipative ratio higher than 0.55 and without leakage of the injectate through the injection channel. Finally, we showed proof of concepts for the successful design of injectable and gellable cellulose nanofiber-filled chitosan formulations with suitable rheological properties for IVD tissue engineering [74].

## 4. Conclusions

Injectable suspensions of cellulose nanofibers dispersed in chitosan solutions were processed and their rheological properties were characterized. The addition of CNF did not alter the injectability of the initial CHI solution. It opens the way to minimally invasive treatments for disc tissue repair/regeneration. The analyses by Couette and capillary rheometry by using an injection setup provided the rheological behavior in an extended range of shear rates, relevant to investigate the injectability of CNF/CHI formulations. The rheological studies revealed two relaxation processes of CHI chains occurring in CHI/CNF suspensions. The slower process (occurring at lower shear rates) was ascribed to CHI chains interacting with CNFs, and the faster process was related to CHI intermolecular interactions and entanglements. By increasing the CNF content, a significant increase of the Newtonian viscosity at low shear rates was observed for CNF content as low as 0.2% (*w*/*w*). The flow at higher shear rates was less affected: CNF/CHI suspensions and CHI solution exhibited practically the same flow exponent. At high shear rates the presence of CNFs seems to be negligible to the rheological behavior, which was also ascribed to the CNF orientation. CNF-filled chitosan hydrogels exhibited an increase of the elastic modulus with the increase of the CNF content. Thus, CHI/CNF interactions like H-bonding and hydrophobic interactions can be tuned to optimize the properties of the composite hydrogels. Studies with CNF contents above 0.8% (*w*/*w*) yielded less reproducible results, which might be due to a bad dispersion and distribution of the nanofibers in the suspensions (“sol state”) and hydrogels.

We proposed the development of injectable cellulose nanofiber-filled chitosan formulations for disc nucleosupplementation. From experiments performed in pig models, we showed that the intradiscal injection and in situ gelation of the formulations resulted in: (i) localization of the implant at the injection site; (ii) restoration of the viscoelastic properties of the discs, which is relevant to restore disc biomechanics; (iii) restoration or increase of the disc height, which is of therapeutic interest to suppress or decrease back pain by avoiding nerve root compression. These CNF/CHI formulations which were aimed at combating mechanical disc failure show promising results as nanofibril-reinforced and non-cellularized bioactive biomaterial to promote disc regeneration.

## Figures and Tables

**Table 1:** Flow exponent index *n_wr_* values of CHI solution and CHI/CNF suspensions in aqueous conditions (CHI concentration: 2% (*w*/*w*)).

Sample	*n_wr_*
CHI2	0.21 ± 0.1
CHI2CNF0.2	0.21 ± 0.1
CHI2CNF0.3	0.23 ± 0.1
CHI2CNF0.4	0.21 ± 0.1

**Table 2:** Flow parameters determined from the extended flow diagrams of CNF/CHI formulations, by using the Cross model (Equation (1)) for the CHI solutions and the double Cross model (Equation (11)) for the CNF/CHI suspensions.

Sample	η_0*, Chi*_ *(Pa.s)*	τ *_Chi_ (s)*	*p_Chi_*	*s*	η_0*, CNF*_ *(Pa.s)*	τ *_CNF_ (s)*	*p _CNF_*
Hydroalcoholic conditions:							
CHI1.7	62	1.0	0.71				
CHI1.7CNF0.3	62	1.0	0.71	0.95	70	3.0	0.99
CHI1.7CNF0.4	62	1.0	0.71	0.95	220	9.5	0.77
							
CHI2	430	4.1	0.78				
CHI2CNF0.2	430	4.1	0.78	0.95	385	8.5	0.93
CHI2CNF0.3	430	4.1	0.78	0.95	591	9.1	0.99
Water conditions:							
CHI2	151	0.8	0.79				
CHI2CNF0.2	151	0.8	0.79	0.97	257	8.3	0.93
CHI2CNF0.3	151	0.8	0.79	0.99	282	8.3	1.54
CHI2CNF0.4	151	0.8	0.79	1.03	343	8.3	1.30

**Table 3:** Compression stress relaxation modeling parameters for different intervertebral discs of a growing-finishing pig: (S) non-injected healthy discs; (I) mechanically tested and then injected discs; and (PI) disc pre-injected before mechanical test. The analysis was performed according to a three branch Maxwell element, according to the relaxation law: $\sigma \left(t\right)=\sigma_{0}+\sigma_{01\cdot }e^{-t/\tau 1}+\sigma_{02\cdot }e^{-t/\tau 2}+\sigma_{03\cdot }e^{-t/\tau 3}$.

Sample	Relax. Frac.*	τ_1_ (s)	τ_2_ (s)	τ_3_ (s)	σ_01_ (MPa)	σ_02_ (MPa)	σ_03_ (MPa)	σ_0_ (MPa)	IVD Type
T8-T9	0.86	394.25	53.43	5.17	0.265	0.160	0.432	0.141	S
T10-T11	0.91	629.51	101.35	7.65	0.217	0.211	0.367	0.074	S
T12-T13	0.86	327.26	63.99	6.69	0.249	0.143	0.307	0.114	S
T14-T15	0.90	605.27	94.24	8.09	0.166	0.140	0.352	0.071	S
L1-L2	0.86	422.44	54.92	7.80	0.187	0.124	0.216	0.086	S
**Mean +/− sd**	**0.88 +/− 0.02**	**475.74 +/− 134.12**	**73.59 +/− 22.61**	**7.08 +/− 1.19**	**0.217 +/− 0.04**	**0.156 +/− 0.03**	**0.335 +/− 0.08**	**0.097 +/− 0.03**	**S**
T8-T9	0.86	423.34	83.35	6.99	0.284	0.147	0.404	0.134	I
T12-T13	0.91	383.18	62.85	6.71	0.271	0.162	0.312	0.076	I
T14-T15	0.88	328.15	51.44	6.56	0.209	0.150	0.291	0.089	I
L1-L2	0.88	429.04	66.29	8.12	0.198	0.119	0.216	0.075	I
**Mean +/− sd**	**0.88 +/− 0.02**	**390.93 +/− 46.56**	**65.99 +/− 13.20**	**7.09 +/− 0.70**	**0.241 +/− 0.04**	**0.144 +/− 0.02**	**0.306 +/− 0.08**	**0.095 +/− 0.03**	**I**
T9-T10	0.92	370.91	72.19	6.48	0.256	0.234	0.326	0.069	PI
T11-T12	0.92	353.52	64.99	6.27	0.221	0.188	0.314	0.061	PI
T13-T14	0.92	352.23	55.87	6.36	0.182	0.159	0.315	0.056	PI
T15-L1	0.92	381.10	56.11	6.45	0.170	0.161	0.294	0.057	PI
**Mean +/− sd**	**0.920 +/− 0.02**	**364.44 +/− 14.00**	**62.29 +/− 7.85**	**6.39 +/− 0.09**	**0.207 +/− 0.04**	**0.185 +/− 0.03**	**0.312 +/− 0.01**	**0.061 +/− 0.01**	**PI**

* Relaxable fraction = (σ_01_ + σ_02_ + σ_03_)/(σ_0_ + σ_01_ + σ_02_ + σ_03_).

## Supplementary Materials

The following are available online at http://www.mdpi.com/2073-4360/10/11/1202/s1, Figure S1: 2D X-ray synchrotron images (Top) and radial average scattering curves (Bottom) of CNF/CHI composite hydrogels containing 2% (*w*/*w*) of CHI and 0.4% (*w*/*w*) of CNFs. The analyses were performed at a microfocus beamline with beam size of 10 µm (Beamline µSpot, Bessy II, HZB Berlin, Germany) with a scan resolution of 10 µm. Figure S2: Pig and rabbit spine models with examples of ex vivo injection of CNF/CHI formulation in the ventral side of T15-L1 disc of pig model, and of L4-L5 disc of rabbit model. The injections were performed with 25G needles (0.5 × 16 mm, BD Microlance^TM^ 3, BD, Heidelberg, Germany) connected to a 1-mL syringe (BD Luer-Lok™, BD, Heidelberg, Germany); Figure S3: Magnetic resonance imaging (MRI) of a spine pig model, showing the injectate after 1 h of injection ex vivo of 100 µL of gadolinium-enriched 3% (*w*/*w*) CHI formulation. Physiological mechanical loading of the spine in compression, torsion and bending was also performed before the MRI images were taken; Figure S4: Histological analysis (hematoxylin-eosin staining) at Day 95 of the L4-L5 (Left) and L5-L6 (Right) discs of pig model, which were injected with 2% and 3% (*w*/*w*) CHI solutions, respectively.
