# Supplementary material for: Injectable and Gellable Chitosan Formulations Filled with Cellulose Nanofibers for Intervertebral Disc Tissue Engineering
Source: Polymers (Basel). 2018 Oct 27;10(11):1202. doi: 10.3390/polym10111202 (PMC6290636; doi:10.3390/polym10111202)
Supplement: Supplementary file 1 [file polymers-10-01202-s001.zip › Supplementary Material_ Polymers_ b.docx]

Injectable and gellable chitosan formulations filled with cellulose nanofibers for intervertebral disc tissue engineering

Ingo Dönch^1,2^, Maria E.W. Torres-Ramos^1,2^, Alexandra Montembault^3^, Paula Nunes de Oliveira^3^, Celia Halimi^3^, Eric Viguier^4^, Laurent Heux^5^, Robin Siadous^6^, Rossana M.S.M. Thiré^7^, Anayancy Osorio-Madrazo^1,2,^*

^1^ Institute of Microsystems Engineering IMTEK, Laboratory for Sensors, University of Freiburg, 79110 Freiburg, Germany

^2^ Freiburg Materials Research Center FMF, University of Freiburg, 79104 Freiburg, Germany

^3^ Ingénierie des Matériaux Polymères (IMP), CNRS UMR 5223, Université Claude Bernard Lyon 1, Université de Lyon, 15 Bd A. Latarjet, 69622 Villeurbanne Cedex, France

^4^ VetAgro Sup, Veterinary School, University of Lyon, 69280 Marcy l'Etoile, France

^5^ Centre de Recherches sur les Macromolécules Végétales (CERMAV)-CNRS UPR 5301 Université Grenoble-Alpes, 38041 Grenoble, France

^6^ INSERM U1026 Bioingénierie tissulaire, Université Bordeaux, F-33000 Bordeaux, France

^7^ COPPE/Program of Metallurgical and Materials Engineering, Federal University of Rio de Janeiro, 21941-972 Rio de Janeiro, PO Box 68505, Brazil

***** Correspondence: anayancy.osorio@imtek.uni-freiburg.de; Tel.: +49-761-203- 67363

**X-ray synchrotron analysis of the CNF/CHI formulations**

The methodology followed to prepare the formulations of cellulose nanofibers (CNF) dispersed in chitosan (CHI) solutions provided a satisfactory homogenization of the nanofibers in the suspensions, which were stable without any apparent CNF macro-aggregates and allowed preparing CNF-filled CHI composite hydrogels of good dispersion and distribution. 2D X-ray synchrotron analyses (see §2.6 of Materials and Methods) were performed, on different directions of composite hydrogels, at a microfocus beamline with beam size of 10 μm (Beamline μSpot, Bessy II, Berlin) with an scan resolution of 10 μm. Fig. S1 show examples of 2D X-ray synchrotron images and corresponding radial average scattering curves obtained for a hydrogel consisting of CHI 2 % (w/w) and CNF 0.4 % (w/w). In spite the low concentration of CHI and specially of crystalline cellulose nanofibers in the hydrogels, in all X-ray diffraction patterns the reflection (002) of the Cellulose Iα was observed. The major contribution in the diffractogram was the water signal, and it was difficult to observe that of chitosan forming the hydrogel network in a highly hydrated system, where water and crystalline cellulose were more relevant to the diffraction.

|  | 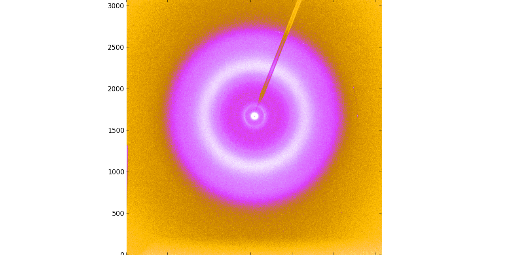 | 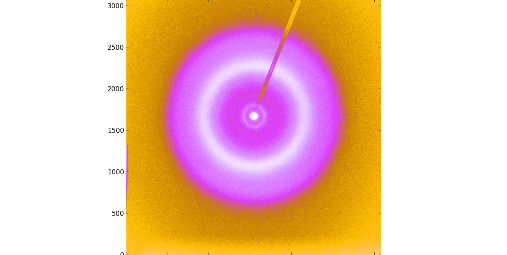 |
| --- | --- | --- |
| 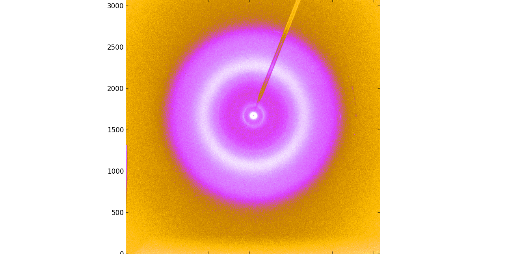 | 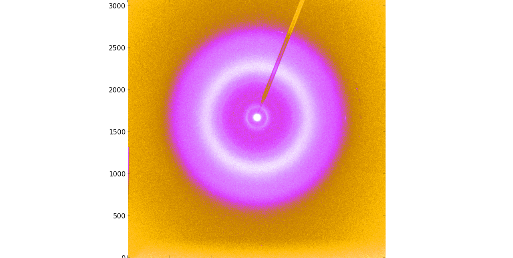 | 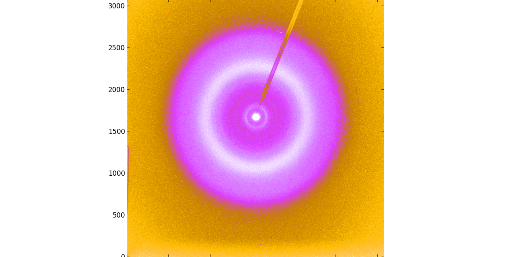 |
| 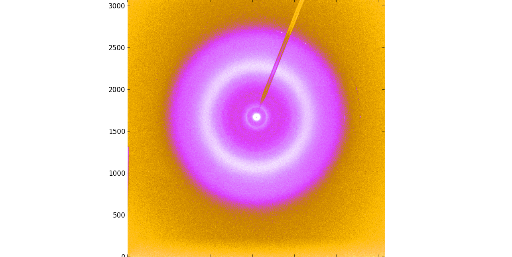 | 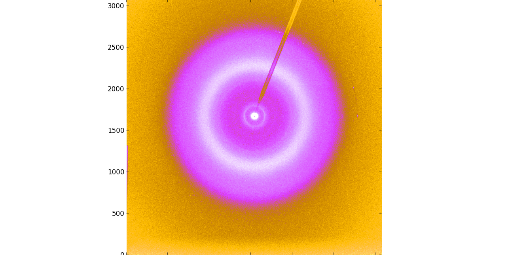 | 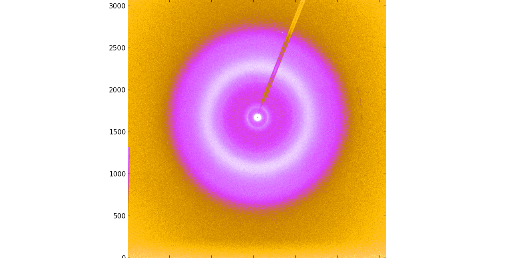 |
| 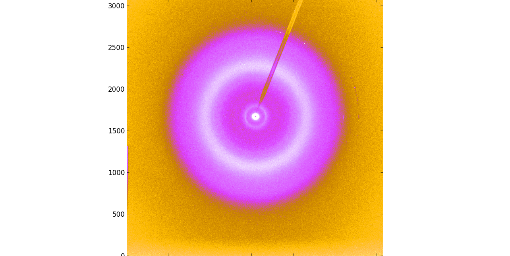 | 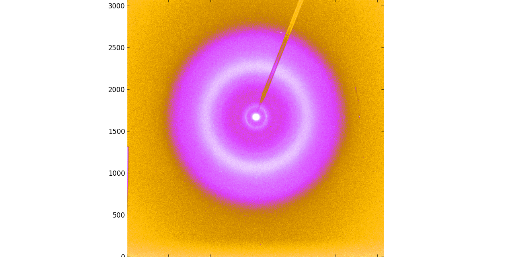 | 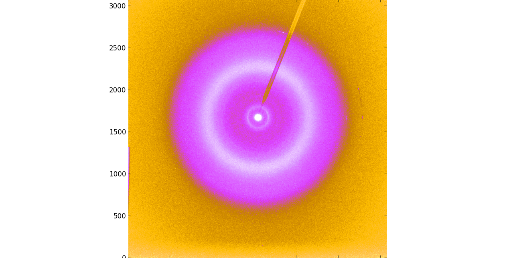 |

**Figure S1.** 2D X-ray synchrotron images (*Top*) and radial average scattering curves (*Bottom*) of CNF/CHI composite hydrogels containing 2 % (w/w) of CHI and 0.4 % (w/w) of CNFs. The analyses were performed at a microfocus beamline with beam size of 10 μm (Beamline μSpot, Bessy II, HZB Berlin, Germany) with an scan resolution of 10 μm.

**Intervertebral discs of animal spine models. Injection of CNF/CHI formulations into the disc nucleus**

**Figure S2.** Porc and rabbit spine models with examples of e*x vivo* injection of CNF/CHI formulation in the ventral side of T15-L1 disc of porc model, and of L4-L5 disc of rabbit model. The injections were performed with 25G needles (0.5 x 16 mm, BD Microlance^TM^ 3, BD, Heidelberg, Germany) connected to a 1 mL syringe (BD Luer-Lok™, BD, Heidelberg, Germany).

**Localization of the injected formulation within the disc. Ex vivo evaluation**

Injections were carried out with needles as thin as the 25G (0.5 x 16 mm). The used needle had smaller diameter than those currently used in clinical intradermal applications, to avoid the lesion of the annulus and possible disc degeneration. Besides, it should reduce the risk of leakage of the injected formulation through the injection canal. Fig S3 shows a magnetic resonance imaging MRI (see § 2. Materials and Methods) of a spine pig model, showing that the injectate of CHI-based formulation stayed localized at the implantation site, even after physiological mechanical loading of the spine in compression, torsion and bending.[60]

**Figure S3.** Magnetic resonance imaging MRI of a spine pig model, showing the injectate after 1 h of injection *ex vivo* of 100 μL of gadolinium-enriched 3 % (w/w) CHI formulation. Physiological mechanical loading of the spine in compression, torsion and bending was also performed before the MRI images were taken. Adapted from [60].

**Histological analyses after in vivo disc injection with the formulation**

|  | 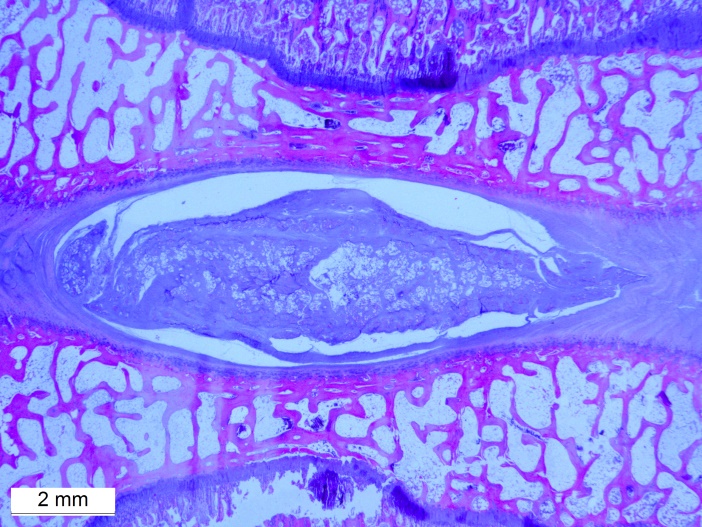 |
| --- | --- |

**Figure S4.** Histological analysis (hematoxylin-eosin staining) at Day 95 of the L4-L5 (*Left*) and L5-L6 (*Right*) discs of pig model, which were injected with 2 and 3 % (w/w) CHI solutions, respectively. Adapted from [60].

**References**

60. Halimi, C.; David, L.; Viguier, E.; Delair, T.; Montembault, A. Chitosan Aqueous Solution for Injection for the Prevention or the Treatment of Intervertebral Disc Degeneration; Patent FR3039402B1; France, 2017.
